# Interactions of flower visitors with bitter gourd (*Momordica charantia* L.) and effects of right target and wrong target flower visits on plant reproduction

**DOI:** 10.1038/s41598-025-20968-w

**Published:** 2025-10-22

**Authors:** Ujjwal Layek, Sourabh Bisui, Prakash Karmakar

**Affiliations:** 1 Department of Botany, Rampurhat College, Rampurhat, 731224 West Bengal India; 2 Department of Botany & Forestry, Vidyasagar University, Midnapore, 721102 West Bengal India

**Keywords:** Ecology, Plant sciences

## Abstract

Studying flower visitors, visitation patterns, and pollinator effectiveness can provide valuable insights into the interactions between plants and pollinators. Bitter gourd (*Momordica charantia*), a key vegetable crop, relies on flower visitors, especially pollinators. However, the specific role of wild pollinators and the impact of different visit types on bitter gourd pollination remain unclear. We aimed to uncover plant–pollinator interactions on the crop species. We recorded the flower visitors and their visitation patterns, categorising visits as right target when the visitor collected floral resources, and as wrong target when the visitor did not collect any resources from the flowers. We also determined the impacts of two types of flower visits on plant reproduction based on pollen deposition and the resulting fruit and seed sets after a single visit by different pollinators. Several insect species visited bitter gourd flowers, while the most abundant and effective pollinators (based on the pollination service index, PS*i*) were *Apis cerana*, *Apis dorsata*, *Apis florea*, *Austronomia ustula*, *Lasioglossum albescens*, *Lasioglossum cavernifrons*, *Lasioglossum funebre*, and *Nomia* (*Hoplonomia*) *elliotii*. Most visitors, especially bees, made both the right target and the wrong target visits to female flowers. In addition to the right target visits, pollinators deposited significant pollen grains on flower stigmas via wrong target visits, leading to fruit and seed sets. These findings highlight the overlooked yet crucial role of wrong target visits in pollination and reproductive success.

**Supplementary Information:**

The online version contains supplementary material available at 10.1038/s41598-025-20968-w.

## Introduction

Flowering plants and flower visitors’ interactions may be mutualistic, commensalism or amensalism^1,2^. Many animals visit flowers, yet their contribution to plant reproduction can vary. Some visitors do not affect plant reproduction or can even be detrimental to pollination by destroying flower structures (mainly florivores)^3–5^, or consuming food resources available for other visitors^6–8^. However, most floral visitors mutually associate with flowering plants, collecting floral rewards and providing pollination services, helping plant reproduction^9–11^.

Visitors sit on flower parts or inflorescences (or some are in continuous flight mode) and collect floral rewards, a visit type known as a right target visit. Another visit type is the wrong target visit, where visitors sit on flowers to collect rewards but leave the flowers without taking the rewards (possibly due to the unavailability or inaccessibility of the flower rewards). Again, right target and wrong target visits of the flower visitors can be classified into two types: (i) legitimate (i.e., visitors touch the reproductive parts of the flowers during their visits), and (ii) illegitimate (i.e., visitors do not touch the reproductive parts of the flowers during their visits).

Some insects that frequently visit flowers include bees, butterflies, flies, moths and wasps^12–14^. Bees are widely regarded as the most important pollinators^15–18^. There are many bees, including honeybees (*Apis* spp.), bumble bees (*Bombus* spp.), solitary bees (e.g., carpenter bees, halictid bees, leafcutter bees, and mason bees), and stingless bees (tribe: Meliponini). Their contribution to flower pollination can be highly variable, as pollination efficiency of a pollinator depends on numerous factors like bees’ floral preferences, foraging activity and behaviour, and flower characteristics^19–21^ and also on environmental conditions^22–24^.

The pollinator contribution can be quantified with several indices, such as pollination intensity (Primack & Silander^25^), single-visit efficiency (Spears^26^), multiple-visit efficiency (Stavert^27^) and pollinator importance (Layek et al.^28^). Among them, frequently used indices are pollination intensity (i.e., the number of pollen grains deposited on the stigmas by a single visit)^25^ and single-visit pollination efficiency index (a method for evaluating the relative importance of different visitors to a plant reproduction by allowing visits to virgin flowers and assess the subsequent fruit or seed sets)^26^.

In agricultural systems, achieving maximum crop yield is essential. However, with the decline of pollinators, many flowering crops experience pollination deficits^29,30^. The quality and quantity of fruits largely depend on effective pollination services^31–34^. Therefore, determining effective pollinators for a crop species is crucial, and the study of crop plant and pollinator interactions remains at the forefront of crop pollination biology^35–37^. It will help manage effective pollinators and provide optimum pollination services.

Bitter gourd (*Momordica charantia* L.) is a member of the plant family Cucurbitaceae and obligately depends on pollinators for fruit set^30^. Concerning the floral visitors of the crop species, a few research works (e.g., Subhakar et al.^38^; Saeed et al.^39^; Yogapriya et al.^40^; Bisui et al.^33^) are available, both from India and outside. Studies on pollinator visitation patterns and plant-pollinator interactions are limited, with most existing research concentrating mainly on floral visitor diversity.

In this study, we aimed to investigate the interactions of pollinators with bitter gourd. We estimated the abundance, diversity and richness of floral visitors. We examined the various types of visits to flowers and their effects on plant reproduction. We investigated the effective pollinators of the bitter gourd by considering pollen deposition on stigmas, fruits, and seed sets in different treatments. In this context, our study addressed the following questions: (1) What are the floral visitors of the crop species? (2) What are the effective pollinators? (3) Do the different types of visitation patterns of pollinators influence plant reproduction differentially?

## Materials and methods

### Plant species

**Fig. 1:**
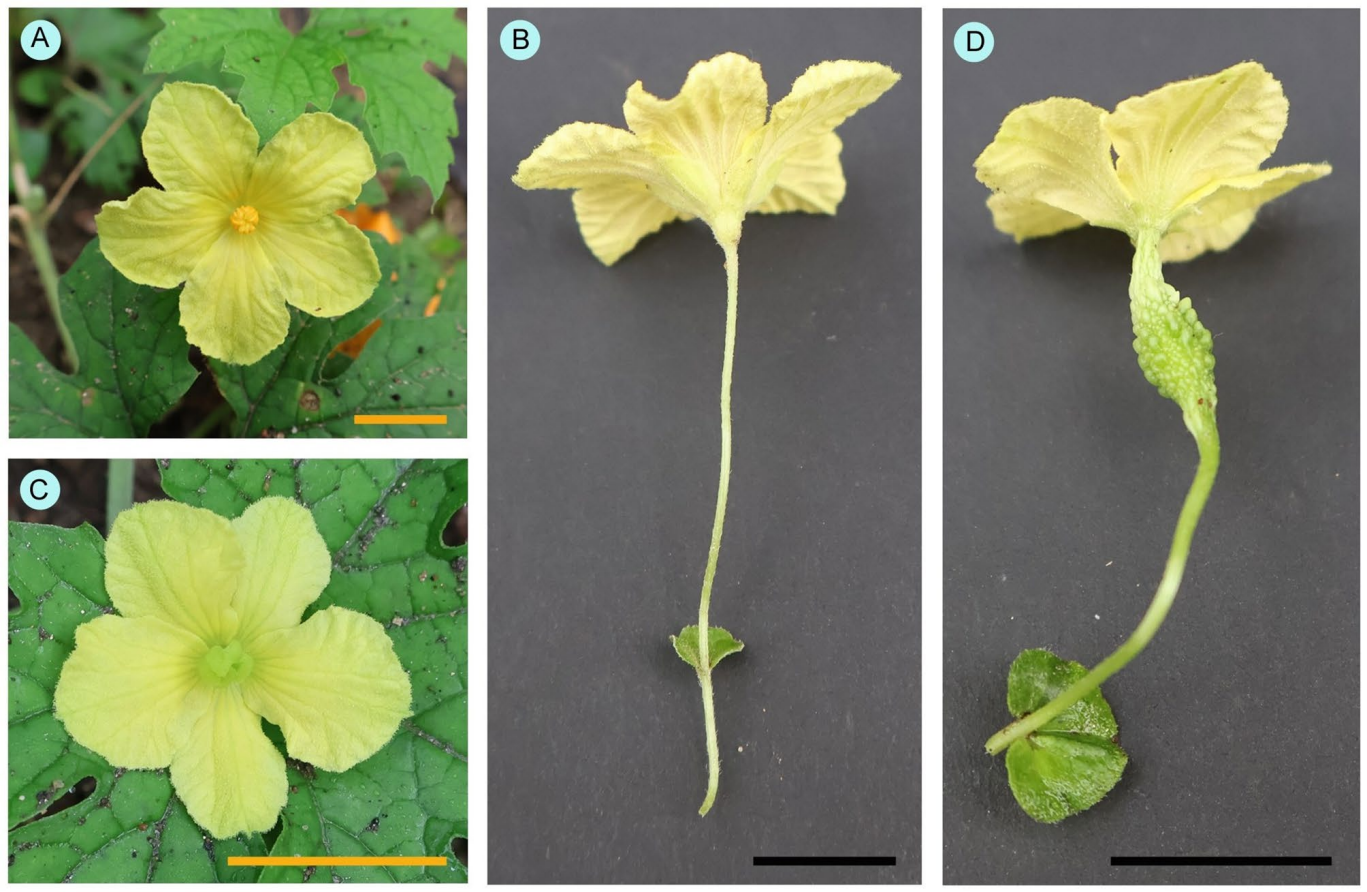
Flowers of *Momordica charantia*. (**A**–**B**) Male flowers and (**C**–**D**) female flowers. Scale bars = 10 mm.

The present study was conducted on a cucurbitaceous crop, *Momordica charantia*. This monoecious plant produces both male and female flowers (Fig. 1) on the same plants. The number of female flowers remained low compared to male flowers; female flowers were only about 16% of total flowers (authors’ observation) and varied from time to time. During peak flowering time, the abundance of flowers (number of flowers/m^2^ area) was about 17.24 ± 4.26.

### Study sites

**Fig. 2:**
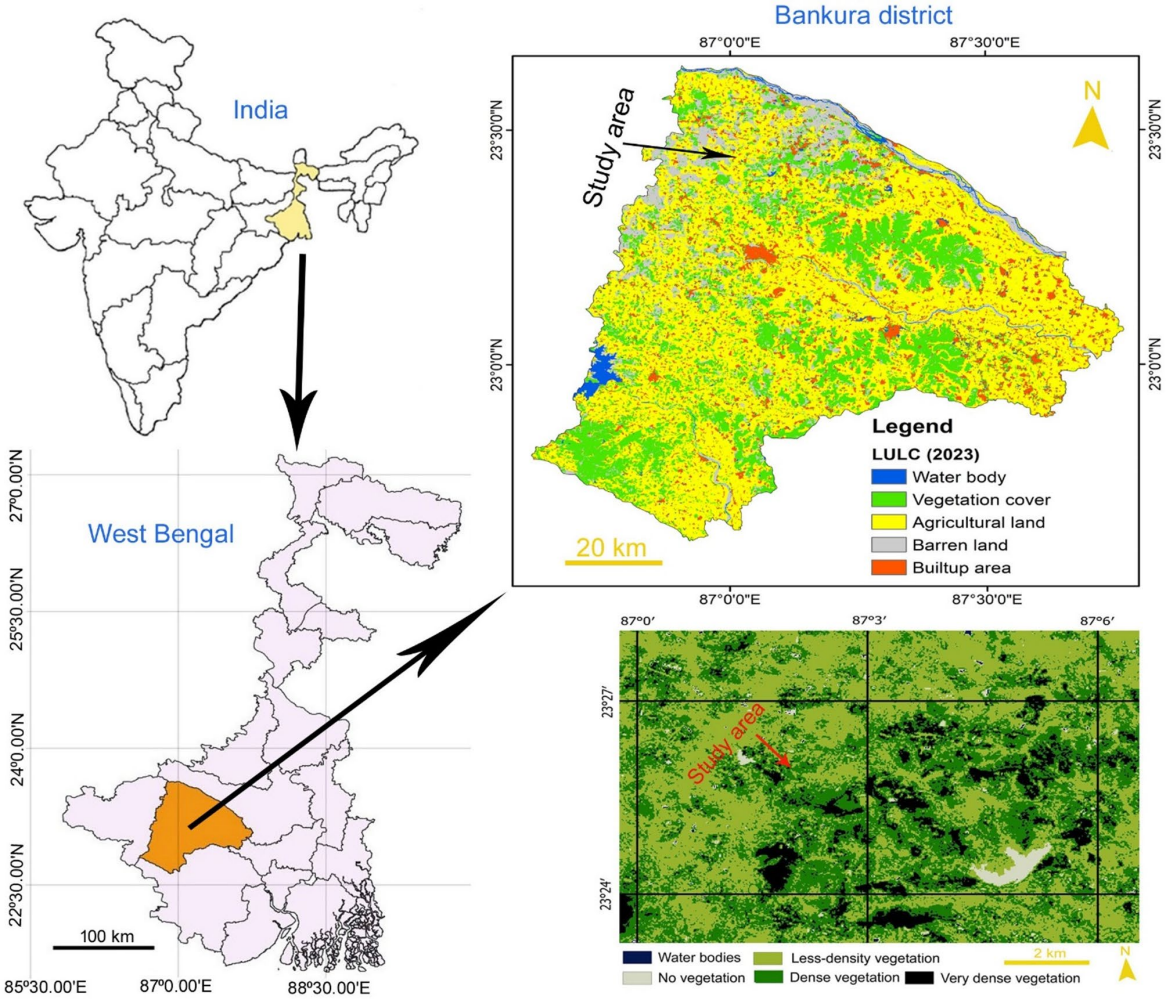
The map shows the study area.

We conducted the present work in open fields (*n* = 5 fields) of farmers at Jenadihi village in Bankura district, West Bengal, India (Fig. 2), during the summer months (April–June) in three consecutive years: 2022, 2023, and 2024. The area is characterised by a mixed vegetation, including trees, bushes, and some agricultural fields. Most fields are rectangular in shape and variable in size, with dimensions of 8.13–15.47 m in length and 6.18–13.04 m in breadth. Some farmers cultivated bitter gourds in a small portion of a field. Within the agricultural zone, a few fields (about 7.84%) were used for bitter gourd cultivation, while others (about 48.04% of the fields) were planted with other crops (such as *Cucumis sativus* L., *Sesamum indicum* L, and *Solanum melongena* L.), and some (about 44.12% of the fields) remained uncultivated at that time. Some trees (e.g., *Borassus flabellifer* L. and *Tectona grandis* L.f.) and shrubs (e.g., *Chromolaena odorata* (L) R. M. Kink & H. Rob. and *Xanthium strumarium* L.) were also there in the ridges of some agricultural fields. During the peak blooming period, the study area confronted a hot, dry summer with a maximum day temperature of 44˚C.

### Traits (attractants) of male and female flowers

We recorded some traits (that may attract flower visitors) of male and female flowers, including flower morphology (e.g., shape, size, and colour), pollen production per flower, nectar yield per flower, and flower volatile organic compounds (VOCs). Pollen grains were counted using a compound microscope (Primo Star, Zeiss) with a known volume (i.e., 10 µL) of pollen solution, which was taken with a micropipette. A detailed methodology is provided in Table S1. Nectar yield per flower was estimated using 5 µL micro capillary tubes (Table S1). Flower VOCs were analysed directly using Headspace GC-MS (Trace 1300 Gas Chromatography, Thermo Fisher Scientific and ISQ QD Single Quadrupole Mass Spectrometer, Thermo Fisher Scientific) (see Table S1 for the methodologies).

### Floral visitors

The survey was conducted over the peak flowering season of bitter gourd (mid-April–mid-May), during daytime, which was divided into seven two-hour time slots (from 4:00 to 18:00 h). We employed a direct observation method (*N* = 40 × 7 = 280 observations, 40 observations per time slot, and seven time slots), where each survey (i.e., field-based sampling) was conducted for 5 min on an area of 1 m² within the crop fields. The encountered flower visitors were recorded. Most visitors were identified in the field, and unidentified flower visitors (one individual per species) were captured using an insect net for later identification.

We estimated the abundance (i.e., the number of individuals of a species/m^2^ area/5 min) of each flower-visiting species. Then, the relative abundance (RA) of each insect species was calculated as follows (Layek et al.^21^):

$$\:\text{R}\text{A}\:\left(\text{\%}\right)=\frac{\text{n}i}{\text{N}}\times\:100$$

Where n*i* is the number of recorded individuals of the insect species *i*, and N is the total number of recorded individuals for all the flower visitors.

The richness of the flower-visiting community was calculated using the index (D) of Margalef^41^, as follows:

$$\:\text{D}=\frac{\text{S}-1}{{ln}\text{N}}$$

Here, S is the number of flower-visiting species, and N is the total number of individuals. The natural logarithm is denoted by *ln*. The D was calculated for each sample. Here, one survey (i.e., a 5-minute observation on a 1 m² field area) was considered a sample.

The diversity of flower visitors was estimated using the diversity index (*H’*) of Shannon and Weaver^42^ as follows:

$$\:{H}^{{\prime\:}}=-{\sum\:}_{i}^{n}\left(\text{p}\text{i}.{ln}\text{p}\text{i}\right)$$

Here, pi is the proportion of each visitor species within the sample. This proportion is calculated as ni/N, where ni is the number of individuals recorded for *i* species, and N is the total number of individuals recorded in the sample.

### Flower visitation pattern

We recorded the number of visits received for male and female flowers per 5-minute duration. Data were taken for all seven time slots (*N* = 140 observations for male and 140 for female flowers).

We recorded the different types of visits made by visitors to male and female flowers, including (i) right target visit (i.e., a visitor successfully collects floral resources on that visit) and (ii) wrong target visit (i.e., a visitor does not collect floral resources on that visit). Additionally, the legitimacy of a visit was also considered, with legitimate visits involving visitors touching reproductive parts, and illegitimate visits not involving visitors touching reproductive parts. For each type of flower (i.e., male and female), we recorded 20 visits per observation (randomly selected and not limited to a specific insect species). Ten observations were conducted in each time slot, totalling 70 observations per flower type (10 × 7 = 70 observations for each flower type). This resulted in 1,400 recorded visits for male flowers and 1,400 for female flowers (*N* = 20 × 10 × 7 = 1,400 visits for each flower type).

We recorded the types of floral resources (e.g., nectar, pollen grains, and floral tissues) collected by the visitors. We recorded the foraging rate (i.e., flower visitation rate) as the number of flowers visited within a 1-minute duration (*n* ≤ 20 observations for an insect species; for abundant flower-visiting species, *n* = 20 observations per time slot). For visitors with a very low visitation rate (e.g., butterflies, flies, and stingless bees), we counted the flowers visited within a 5-minute duration. Then, we estimated the number of flowers visited per minute by dividing the total (i.e., counted over 5 min) by 5 (here, *n* = 10 observations for an insect species). The flower handling time of visitors was estimated as the amount of time spent per visit on a flower. We conducted ≤ 20 observations for an insect species (for abundant flower-visiting species, *n* = 20 observations per time slot, resulting in *N* = 20 × 7 = 140 observations for an insect species).

On an observation day, we counted the number of male and female flowers within a small area (2 m × 2 m) of the field. Then, we counted the number of visits to both male flowers and female flowers by a focal flower-visiting species. Such ten observations were done for an insect species. Then, we estimated the flower sex type selection index (FS*i*) with a slight modification of Bisui et al.^33^ as follows:

$$\:\text{F}\text{S}i=\frac{\text{V}\text{f}\times\:\text{T}\text{m}}{\text{V}\text{m}\times\:\text{T}\text{f}}$$

Vf is the number of visited female flowers by the insect species *i*, Vm is the number of visited male flowers for the insect species, Tf is the total number of female flowers within the specified area, and Tm is the total number of male flowers within the field area. The value of FS*i* ranges from 0 to ∞. A value of FS*i* greater than 1 indicates a high preference for female flowers over male flowers, and FS*i* < 1 indicates a low preference for female flowers.

### Pollen deposition on stigmas

To measure the amount of pollen deposition on the stigma surface by a single visit by the flower visitors, we caged a small part (2 m × 2 m) of the selected fields by placing nylon nets (with small perforations about 1 mm × 1 mm) over the plants during the peak flowering season of 2024 in the evening. The following morning (7:00–10:00 h, when the flowers opened), the flowers were uncaged, and a researcher stood nearby, observing the female flowers until each received one insect visit. Visitor species and visitation type (i.e., right target or wrong target) were recorded. We then collected the stigmas of the flowers (*n* = 20 flower stigmas per visitor species; limited to the abundant pollinator species) into 1.5 mL Eppendorf tubes containing 1 mL of 70% ethanol. We added one drop of 1% aniline blue to the stigma-containing tubes in the laboratory. After two hrs., the stigma was taken on a slide and flattened. All pollen grains on each slide were counted using a compound light microscope. Pollen that dropped off the stigma during storage was also counted by taking a known volume of pollen solution (10 µL) on a slide and counting the pollen grains.

### Pollination strategies of flower visitors

We observed insect visits (*N* = 1000 visits) to bitter gourd flowers and recorded the number of visits made by each insect species. Based on these counts, we calculated the flower visit proportion (FV) for each species using the following method:

$$\:\text{F}\text{V}=\frac{\text{V}i}{\text{V}t\:}$$

Here, V*i* represents the number of visits made by species *i*, and V*t* denotes the total number of visits made by all species combined.

We studied the pollen adhering sites of visitors using a stereo microscope (Stemi 508, Zeiss) and a scanning electron microscope (Merlin, Zeiss). For this purpose, we caught the flower-visiting insects with a medium-sized plastic container and froze the specimens to immobilise them, then dried them in a hot air oven (Digilab). For the SEM analysis, insect body parts were mounted on metal stubs using sticky carbon tape to enhance conductivity. To prevent charge buildup on the specimen surface, a 10 nm layer of gold nanoparticles was applied as a conductive coating using the Q150R ES sputter coater (Quorum Technologies, UK). The samples were examined using a Zeiss Merlin field-emission scanning electron microscope (FEG-SEM) (Gemini, India). High-vacuum imaging of insect body parts (including adhered pollen) was conducted at magnifications ranging from 1,000X to 40,000X. A secondary electron detector was used for imaging, operating at an acceleration voltage of 5.0 kV.

For the legitimate visitors, we observed the different modes of pollination (according to Layek et al.^43^)― (i) nototribic (pollen transfer to stigma via the dorsal side of the visitor), (ii) sternotribic (pollen transfer to stigma via the ventral side of the visitor), (iii) noto-sternotribic (pollen transfer to stigma via both the dorsal and ventral sides of the visitor), and (iv) appendages mediated (pollen transfer to stigma via the delicate body parts of the visitor, including antennae, proboscis, legs, etc.).

We conducted various pollination treatments, including open pollination, pollinator exclusion, and single-visit experiments. For open pollination, we marked female flowers (*n* = 100) to collect data (i.e., fruit and seed sets). For the pollinator exclusion experiment, we bagged female flower buds (*n* = 50) before they opened and maintained this condition for up to 2 days, resulting in complete loss of stigmatic receptivity. For the single-visit experiments, we caged a small field area as described earlier for single-visit pollen deposition. After taking 20 flower stigmas for each abundant insect species, the remaining flowers that received a single visit (see Table S2) were individually tagged and re-bagged for up to 2 days. All fruits derived from the selected flowers in different experiments were collected after ten days^33^, and the number of seeds was determined. Considering seed sets in all three treatments (single-visit, open pollination, and pollinator exclusion), we estimated the single-visit pollination efficiency index (PE*i*) of dominant visitors (according to Spears^26^) as follows:

$$\:\text{P}\text{E}i=\frac{\text{P}i-\text{Z}}{\text{U}-\text{Z}}$$

Here, P*i* is the mean number of seed sets resulting from a single visit of species *i*; Z is the mean number of seed sets in the pollinator exclusion treatment, and U is the mean number of seed sets obtained in open pollination (i.e., unrestricted visitation).

To judge the effective pollinators of the plant species, we calculated a combined parameter, the pollination service index (PS*i*), by considering the numeric values of multiple parameters (each parameter had a value range of 0 to 1) as follows:

$$\:\text{P}\text{S}i=\text{F}\text{V}\times\:\text{F}\text{S}i\times\:\text{A}\text{R}\:\times\:\text{S}\text{R}$$

Here, FV is the flower visit proportion of species *i*. FS*i* is the flower sex type selection index. For hermaphrodite flowers, each visitor has an FSi value of 1. If a visitor’s FS*i* exceeds 1, the values are rescaled between 0 and 1, with the highest value set to 1 and others adjusted proportionally. AR is anther contact rate (i.e., number of visits contacted with flower anthers divided by the total number of visits; in the case of unisexual flowers, total visits considered only for staminate flowers), and SR is the stigma contact rate (i.e., number of visits contacted with flower stigma divided by the total number of visits; in the case of unisexual flowers, total visits considered only for pistillate flowers). The value of the PS*i* for flower visitors ranges from 0 to 1, with higher values indicating more effective pollinators for the plant species.

### Fruit and seed sets in the right target and the wrong target visits

We measured the fruit and seed sets in various pollination treatments, including single-visit experiments with the right target and wrong target visits. The methodologies for conducting pollination treatments were already mentioned in the previous section. After ten days of flower opening, we collected the fruits derived from the right target and wrong target visits (considering a single visit), and the number of seeds was determined. We estimated the percentage of fruit set by considering the total number of selected flowers on a sampling day for each treatment type, and seed set as the number of seeds derived from each flower.

### Data analysis

We evaluated the data within each group to check key assumptions required for parametric tests, including normality (assessed using the Shapiro-Wilk test and Q-Q plots), homoscedasticity (using scatter plots and the Breusch-Pagan test), and homogeneity of variances (using Levene’s test). If the assumptions are fulfilled, then we performed parametric tests like an independent-sample t-test (for two groups, e.g., fruit set derived from right target and wrong target visits for an insect species), and a one-way ANOVA test (for greater than two groups, e.g., fruit set resulting from a single visit by different insect species). In the case when the assumptions are not fulfilled, we performed non-parametric tests like the Mann-Whitney test (for two groups, e.g., the number of visits received by male and female flowers, right vs. wrong target visit on pollen deposition and seed set) and Kruskal-Wallis H test (for greater than two groups, e.g., abundance, richness, diversity of visitors, flower visitation rate and handling time, single-visit pollen deposition and seed set for different insect species). When the p-value was significant (i.e., *p* ≤ 0.05) for tests involving more than two groups, we conducted post-hoc analyses— Dunn’s test following the Kruskal-Wallis test and Duncan’s multiple range test following one-way ANOVA. We used IBM SPSS Statistics version 26.0 for the statistical analyses.

## Results

### Traits (attractants) of male and female flowers

The male and female flowers of *Momordica charantia* were saucer-shaped. Male flowers were slightly larger than female flowers (Table 1). Male flowers were yellow, while female flowers were greenish yellow. Male flowers had higher flower rewards (e.g., pollen grains and a significant amount of nectar) than female flowers, which secreted a little amount of nectar (0.44 ± 0.18 µL per flower). Both male and female flowers emitted a wide range of volatile organic compounds (Table S3). A few of them (about 43.59% of the compounds) were common to both male and female flowers. The abundant VOCs varied between male and female flowers (Table 1).

**Table 1:** Traits (attractants for visitors) of male and female flowers of *Momordica charantia*.

Flower traits	Male flowers	Female flowers
Morphology		
• Flower shape	Saucer	Saucer
• Flower size (diameter)	31.20 ± 1.51 mm	14.40 ± 2.78 mm
• Colour (petal)	Yellow	Greenish yellow
Pollen yield/flower	9273.33 ± 574.74	0
Nectar yield/flower	2.86 ± 0.30 µL	0.44 ± 0.18 µL
Abundant volatile organic compounds (VOCs)	β-Copaene; 17-(1,5-Dimethylhexyl)-10,13-dimethyl-3-styryl hexadecahydrocyclopenta [a] phenanthren-2-one; Hematoporphyrin; Hexasiloxane, 1,1,3,3,5,5,7,7,9,9,11,11-dodecamethyl;3’H-Cycloprop (1,2) cholesta-1,4,6-trien-3-one, 1’-carboethoxy-1’-cyano-1á,2á-dihydro; Lycopene, 1,1’,2,2’-tetrahydro-1,1’-dimethoxy-, all-trans	Cyclohexasiloxane, dodecamethyl; Ethyl iso-allocholate; Hexasiloxane, 1,1,3,3,5,5,7,7,9,9,11,11-dodecamethyl; Morphinan-4,5-epoxy-3,6-di-ol, 6-[7-nitrobenzofurazan-4-yl] amino

### Floral visitors

**Fig. 3:**
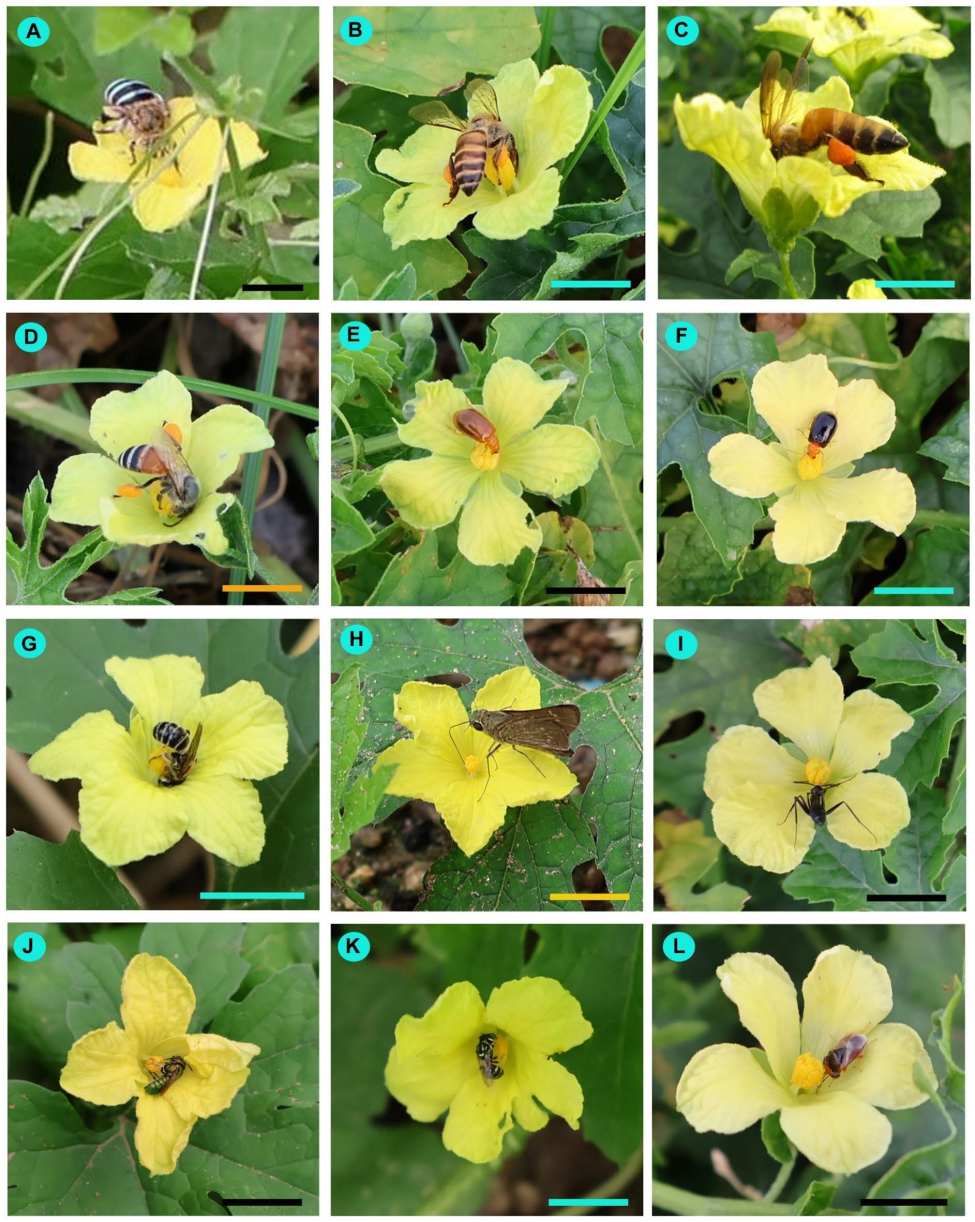
Floral visitors of *Momordica charantia* in West Bengal, India. (**A**) *Amegilla zonata*, (**B**) *Apis cerana*, (**C**) *Apis dorsata*, (**D**) *Apis florea*, (**E**) *Aulacophora foveicollis*, (**F**) *Aulacophora frontalis*, (**G**) *Austronomia ustula*, (**H**) *Borbo cinnara*, (**I**) *Camponotus parius*, (**J**) *Ceratina binghami*, (**K**) *Ceratina hieroglyphica*, (**L**) *Geocoris ochropterus*. Scale bars = 10 mm.

**Fig. 4:**
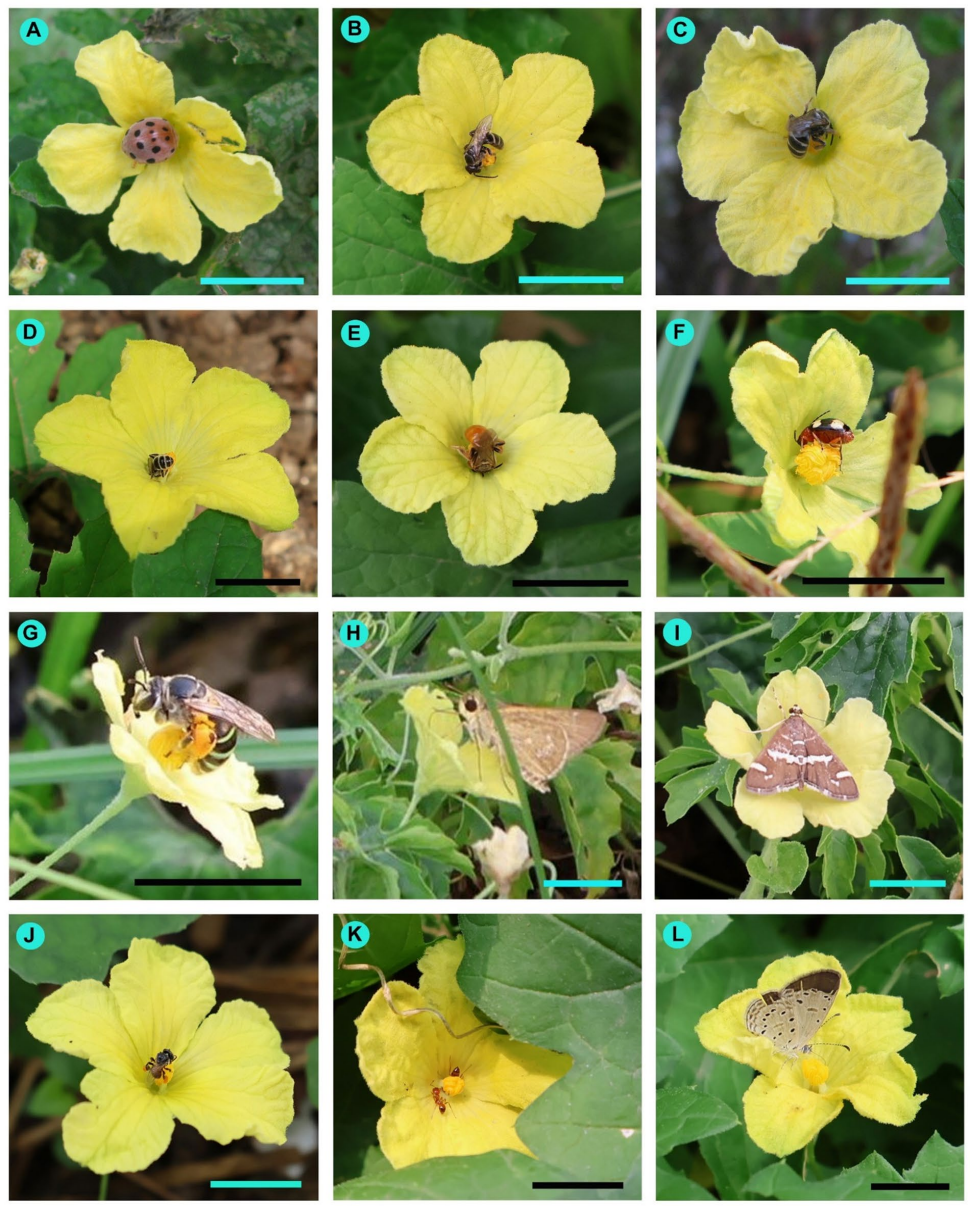
Floral visitors of *Momordica charantia* in West Bengal, India. (**A**) *Henosepilachna septima*, (**B**) *Lasioglossum albescens*, (**C**) *Lasioglossum cavernifrons*, (**D**) *Lasioglossum funebre*, (**E**) *Lasioglossum ovaliceps*, (**F**) *Monolepta signata*, (**G**) *Nomia* (*Hoplonomia*) *elliotii*, (**H**) *Pelopidas sinensis*, (**I**) *Spoladea recurvalis*, (**J**) *Tetragonula pagdeni*, (**K**) *Trichomyrmex destructor*, (**L**) *Zizula hylax*. Scale bars = 10 mm.

In the present study, a total of 45 insect species were recorded as floral visitors of bitter gourd in West Bengal, India (Table S4, Figs. 3 and 4). The most represented insect orders were Hymenoptera (20 species) and Lepidoptera (17 species), followed by Coleoptera (4 species), Diptera (3 species), and Hemiptera (only one species). Among the hymenopteran insects, the majority belonged to the families Halictidae (9 species) and Apidae (7 species). Most butterflies were from the families Nymphalidae (5 species) and Pieridae (5 species).

**Table 2:** Flower visitors and their interactions (e.g., abundance: individuals/m^2^ area/5 min; relative abundance; visitation rate: flowers visited/min; handling time: time spent (sec) per flower/visit; floral resource; and FS*i*- flower sex type selection index) on *Momordica charantia* L. in West Bengal, India.

Floral visitors	Abundance	Relative abundance (%)	Flower visitation rate	Flower handling time	Floral resources	FS*i*
• Coleoptera						
*Aulacophora foveicollis*	0.04 ± 0.19	0.75	0.14 ± 0.05	-	FT	**-**
*Aulacophora frontalis*	0.04 ± 0.24	0.91	0.18 ± 0.08	-	FT	**-**
*Henosepilachna septima*	0.03 ± 0.17	0.60	0.13 ± 0.05	-	FT	**-**
*Monolepta signata*	0.02 ± 0.16	0.53	0.12 ± 0.04	-	FT	**-**
• Diptera						
*Episyrphus balteatus*	0.04 ± 0.20	0.91	0.64 ± 0.31	15.73 ± 13.06	P	0^h^
*Helophilus peregrinus*	0.03 ± 0.18	0.68	0.50 ± 0.22	21.28 ± 18.30	P	-
*Platycheirus albimanus*	0.02 ± 0.16	0.53	0.42 ± 0.20	14.27 ± 11.71	P	-
• Hemiptera						
*Geocoris ochropterus*	0.04 ± 0.23	0.83	0.11 ± 0.03	-	N	-
• Hymenoptera						
*Amegilla zonata*	0.06 ± 0.25	1.28	7.83 ± 1.94	0.84 ± 0.14	N + P	0.46^f^ ± 0.01
*Apis cerana*	0.64 ± 1.12	13.43	15.12 ± 3.27	6.60 ± 3.82	N + P	0.45^f^ ± 0.02
*Apis dorsata*	0.44 ± 0.90	9.36	14.02 ± 3.53	8.68 ± 5.93	N + P	0.46^f^ ± 0.02
*Apis florea*	0.72 ± 1.21	15.85	6.94 ± 2.18	8.64 ± 7.30	N + P	0.47^f^ ± 0.02
*Austronomia ustula*	0.29 ± 0.69	6.11	5.78 ± 1.69	7.35 ± 3.61	N	0.73^a^ ± 0.03
*Braunsapis mixta*	0.02 ± 0.16	0.53	4.47 ± 1.63	11.39 ± 7.14	N + P	0.52^e^ ± 0.02
*Camponotus parius*	0.07 ± 0.34	1.51	0.13 ± 0.05	-	N + P	-
*Ceratina binghami*	0.04 ± 0.22	0.75	4.07 ± 1.66	13.51 ± 7.52	N + P	0.56^d^ ± 0.03
*Ceratina hieroglyphica*	0.02 ± 0.15	0.45	4.30 ± 1.73	12.85 ± 7.33	N + P	0.54^de^ ± 0.02
*Crematogaster laestrygon*	0.04 ± 0.24	0.91	0.11 ± 0.03	-	N	-
*Lasioglossum albescens*	0.38 ± 0.78	8.00	6.06 ± 1.75	6.67 ± 3.70	N	0.74^a^ ± 0.03
*Lasioglossum cavernifrons*	0.50 ± 0.96	10.49	4.85 ± 1.90	12.78 ± 12.37	N + P	0.72^ab^ ± 0.03
*Lasioglossum funebre*	0.39 ± 0.74	8.23	5.86 ± 1.98	7.62 ± 4.64	N + P	0.71^ab^ ± 0.03
*Lasioglossum ovaliceps*	< 0.01	0.15	4.63 ± 1.73	11.18 ± 7.04	N + P	0.59^cd^ ± 0.03
*Nomia* (*Hoplonomia*) *elliotii*	0.30 ± 0.69	6.26	7.36 ± 2.17	7.64 ± 3.89	N + P	0.60^c^ ± 0.03
*Nomia* (*Curvinomia*) *strigata*	0.14 ± 0.34	2.87	7.14 ± 2.13	7.72 ± 3.95	N + P	0.61^c^ ± 0.03
*Scolia soror*	0.02 ± 0.15	0.45	4.75 ± 1.84	5.37 ± 2.08	N	-
*Tetragonula pagdeni*	0.08 ± 0.33	1.66	0.70 ± 0.25	21.82 ± 10.07	N + P	0.42^g^ ± 0.03
*Thyreus nitidulus*	0.03 ± 0.17	0.60	5.72 ± 1.61	7.35 ± 3.14	N	0.42^g^ ± 0.02
*Trichomyrmex destructor*	0.08 ± 0.42	1.74	0.11 ± 0.03	-	N	-
• Lepidoptera						
*Anthene lycaenina*	0.01 ± 0.10	0.23	0.36 ± 0.16	20.47 ± 14.28	N	0.52^e^ ± 0.02
*Appias libythea*	0.01 ± 0.12	0.30	0.42 ± 0.26	12.52 ± 9.27	N	0.54^de^ ± 0.02
*Borbo cinnara*	< 0.01	0.15	1.12 ± 0.66	5.76 ± 4.02	N	-
*Catopsilia pomona*	< 0.01	0.15	0.38 ± 0.18	9.86 ± 8.15	N	-
*Danaus chrysippus*	0.01 ± 0.10	0.23	0.34 ± 0.16	10.38 ± 7.39	N	0.54^de^ ± 0.02
*Euploea core*	< 0.01	0.15	0.30 ± 0.14	8.92 ± 6.35	N	-
*Eurema blanda*	0.01 ± 0.12	0.30	0.42 ± 0.15	15.08 ± 11.71	N	0.63^bc^ ± 0.03
*Eurema hecabe*	0.02 ± 0.15	0.45	0.40 ± 0.14	15.93 ± 11.84	N	0.62^bc^ ± 0.02
*Junonia almana*	< 0.01	0.15	0.32 ± 0.14	10.70 ± 8.46	N	-
*Junonia atlites*	< 0.01	0.15	-	-	N	-
*Junonia iphita*	< 0.01	0.08	-	-	N	-
*Leptosia nina*	< 0.01	0.15	-	-	N	-
*Papilio polytes*	< 0.01	0.08	-	-	N	-
*Pelopidas sinensis*	0.01 ± 0.12	0.30	0.62 ± 0.36	8.14 ± 6.27	N	0.66^b^ ± 0.03
*Spoladea recurvalis*	< 0.01	0.15	-	-	N	-
*Suastus gremius*	0.02 ± 0.15	0.45	0.58 ± 0.36	10.18 ± 7.92	N	0.64^bc^ ± 0.03
*Zizula hylax*	< 0.01	0.08	-	-	N	-

FT-floral tissue, N-nectar, P-pollen. Values are presented as mean ± standard deviation. Superscript letters that differ within a column (i.e., among species) indicate statistically significant differences based on Dunn’s post hoc test at the 0.05 significance level.

**Fig. 5:**
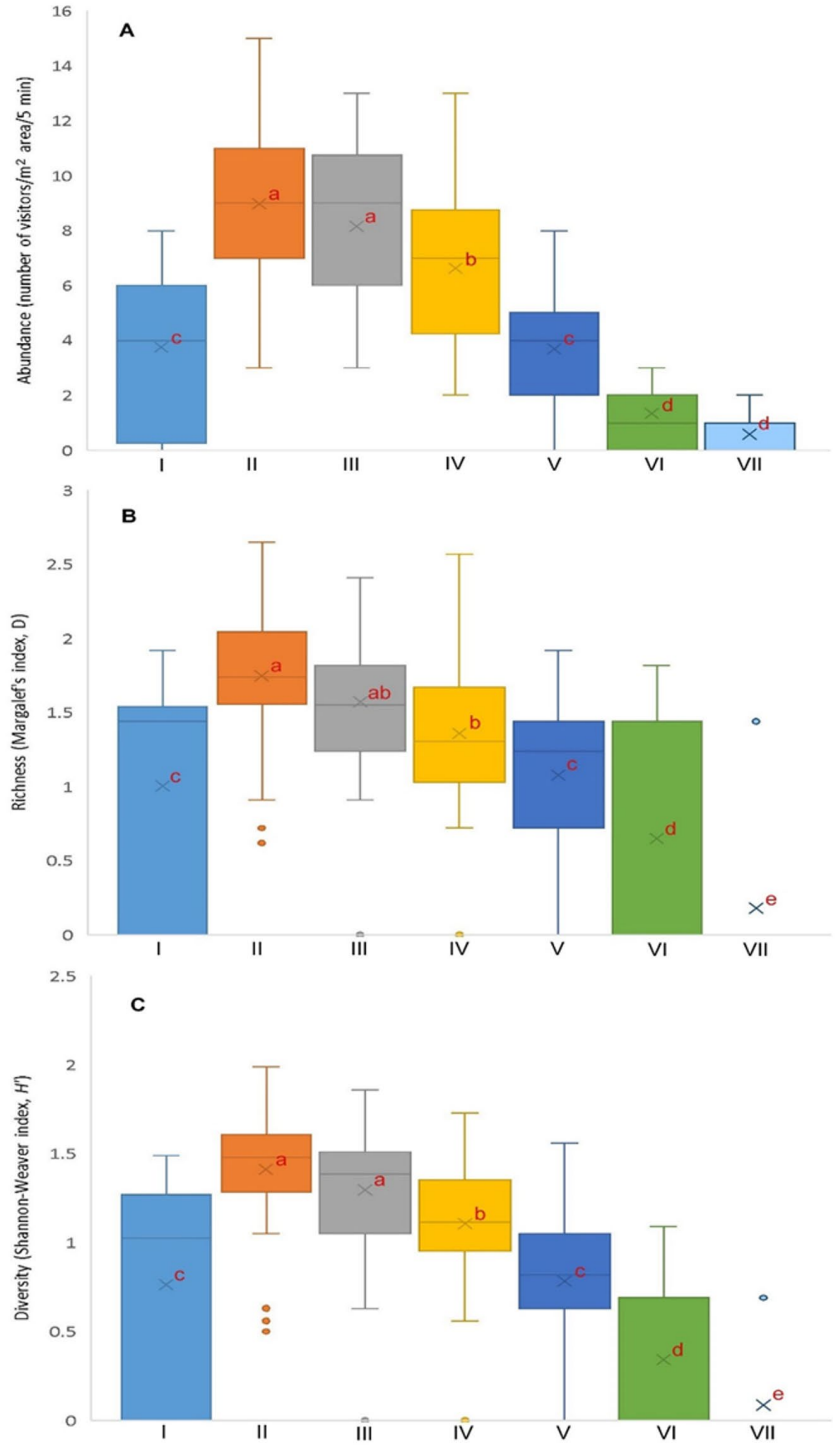
(**A**) Abundance, (**B**) richness, and (**C**) diversity of floral visitors of *Momordica charantia* in West Bengal, India. Different letters associated with means indicate significant differences. I- 4:00–6:00 h, II- 6:00–8:00 h, III- 8:00–10:00 h, IV- 10:00–12:00 h, V- 12:00–14:00 h, VI- 14:00–16:00 h, VII- 16:00–18:00 h.

The abundance of total visitors was 4.73 ± 3.84 visitors/m^2^/5 min. The abundant flower-visiting species were *Apis florea* (0.72 ± 1.21 visitors/m^2^/5 min; relative abundance = 15.85%), *Apis cerana*, *Lasioglossum cavernifrons*, *Apis dorsata*, *Lasioglossun funebre*, *Lasioglossum albescens*, *Nomia* (*Hoplonomia*) *elliotii*, and *Austronomia ustula* (Table 2). The abundance of total visitors varied significantly by daytime (Kruskal-Wallis H = 180.42, *p* < 0.001, df = 6), with a higher abundance recorded at 6:00–10:00 h and lower in the late afternoon (14:00–18:00 h) (Fig. 5A). Considering individual flower-visiting species, their abundance varied diurnally (Table S5), with a higher abundance during 6:00–10:00 h. Sample-wise (5 min observation on 1 m^2^ area), insect species richness (the index of Margalef, D) and diversity (the index of Shannon-Weaver, *H*’) were 1.08 ± 0.76 and 0.83 ± 0.60, respectively. Both the richness and diversity of visitors varied according to the daytimes (richness: Kruskal-Wallis H = 113.45, df = 6, *p* < 0.001; diversity: Kruskal-Wallis H = 152.54, df = 6, *p* < 0.001), with higher values were recorded between 6:00 and 8:00 h, and lower values were observed in the late afternoon (Fig. 5B, C).

### Flower visitation pattern

The number of visits received by a flower per unit of time differed between male and female flowers (Mann-Whitney U = 8239, z = − 2.40, *p* < 0.05). Male flowers received a more significant amount of visits (1.41 ± 1.35 visits/flower/5 min) than female flowers (1.01 ± 1.09 visits/flower/5 min). The number of visits received a flower varied according to daytimes (male flower: Kruskal-Wallis H  = 59.03, df = 6, *p* < 0.001; female flower: Kruskal-Wallis H = 38.97, df = 6, *p* < 0.001). More visits were received during 6:00–10:00 h (Table S6).

**Fig. 6:**
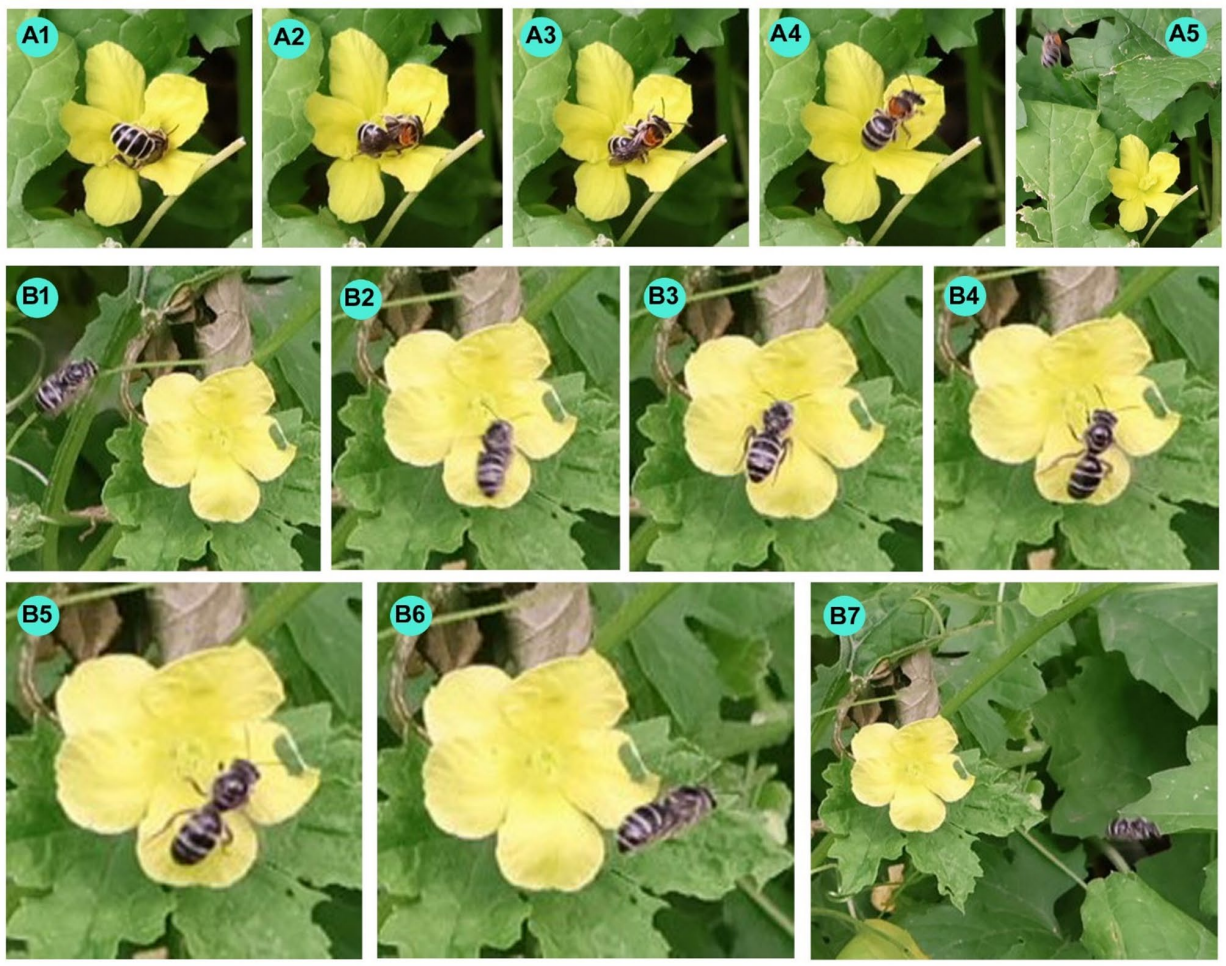
Two types of visits to the flowers of *Momordica charantia* in West Bengal, India. (**A1**–**A5**) A right target visit, and (**B1**–**B7**) a wrong target visit. In both cases, visitors touched the stigma surfaces legitimately.

The male and female bitter gourd flowers received the right target and wrong target visits (Fig. 6). The female flowers received more wrong target visits (43.79 ± 12.38%) than male flowers (Table 3). However, the percentage of wrong target visits on female flowers varied according to daytimes (F_6,63_ = 2.68, *p* < 0.05). On wrong target visits, visitors performed both legitimate and illegitimate types of visits. On average, among the wrong target visits to female flowers, about 49.13 ± 9.86% were of the legitimate type of visit.

The insect visitors collected floral nectar, pollen grains, and floral tissues. The coleopteran members collected floral tissues (acted as florivores). Dipteran flies collected pollen grains only. Hemipteran and lepidopteran members collected nectar only. Among hymenopteran members, ants, wasps and some bees (e.g., *Lasioglossum albescens*, and *Thyreus nitidulus*) collected nectar only, and some other bees (e.g., *Amegilla zonata*, *Apis* spp., *Lasioglossum cavernifrons*, *Lasioglossum funebre*, *Nomia* (*Curvinomia*) *strigata*, *Nomia* (*Hoplonomia*) *elliotii*, and *Tetragonula pagdeni*) collected both nectar and pollen grains (Table 2).

**Table 3:** Different types of visits (right target: collecting flower rewards; wrong target: without flower resource collection; legitimate: touching reproductive parts; illegitimate: without touching reproductive parts) on flowers (%) of *Momordica charantia* L. in West Bengal, India.

Daytime	Male flowers		Female flowers			
	Right target	Wrong target	Right target	Wrong target	Among the wrong target visits	
					Legitimate	Illegitimate
4:00–6:00 h	92 ± 6.32	8 ± 6.32	66.50 ± 9.44	33.50 ± 9.44	49.24 ± 8.65	50.76 ± 8.65
6:00–8:00 h	88.50 ± 5.80	11.50 ± 5.80	60.50 ± 13.43	39.50 ± 13.43	53.36 ± 11.56	46.64 ± 11.56
8:00–10:00 h	87 ± 7.15	13 ± 7.15	54.50 ± 13.63	45.50 ± 13.63	48.74 ± 10.26	51.26 ± 10.26
10:00–12:00 h	84 ± 7.38	16 ± 7.38	52 ± 11.60	48 ± 11.60	45.66 ± 7.46	54.34 ± 7.46
12:00–14:00 h	81.50 ± 8.51	18.50 ± 8.51	48.50 ± 9.73	51.50 ± 9.73	46.37 ± 10.51	53.63 ± 10.51
14:00–16:00 h	83 ± 8.56	17 ± 8.56	53.50 ± 11.32	46.50 ± 11.32	52.28 ± 11.22	47.72 ± 11.22
16:00–18:00 h	85.50 ± 6.85	14.50 ± 6.85	58 ± 11.11	42 ± 11.11	48.25 ± 9.23	51.75 ± 9.23
Throughout daytime	85.93 ± 7.72	14.07 ± 7.72	56.21 ± 12.38	43.79 ± 12.38	49.13 ± 9.86	50.87 ± 9.86

The percentage values are given in mean ± standard deviation.

The flower visitation rate remained high for *Apis cerana* (15.12 ± 3.27 flowers per minute) and *Apis dorsata* (14.04 ± 3.53 flowers per minute). The visitation rate was meagre for butterflies, flies, and stingless bees. The visitation rate significantly differed according to daytime for the most abundant insect species (e.g., *Apis cerana*: Kruskal-Wallis H = 18.54, df = 6, *p* < 0.01), with comparatively higher rates during 8:00–12:00 h and slightly lower in the early morning and late afternoon (Table S7). The flower handling time (i.e., time spent on a flower per visit) was high for some butterflies (e.g., *Anthene lycaenina*), flies and stingless bees. The flower handling time was very low for *Amegilla zonata* (0.84 ± 0.14 s). For most insect species, the flower handling time varied across daytimes (e.g., *Apis cerana*: Kruskal-Wallis H = 19.54, df = 6, *p* < 0.01), with higher values during the early morning (4:00–8:00 h) and lower during 10:00–14:00 h (Table S8).

The flower sex selection index (FS*i*) values ranged from 0 to 0.74, indicating an overall preference for male flowers over female flowers among all flower-visiting species. The values of FS*i* varied across flower-visiting species (Kruskal-Wallis H = 226.96, df = 23, *p* < 0.001). Some halictid bees [e.g., *Austronomia ustula*, *Lasioglossum albescens*, *Lasioglossum cavernifrons*, *Lasioglossum funebre*, *Nomia (Curvinomia) strigata*, and *Nomia (Hoplonomia) elliotii*], and some butterflies (e.g., *Pelopidas sinensis*, and *Suastus gremius*) showed relatively higher FS*i* values compared to other visitors (Table 2).

### Pollen deposition

Single-visit pollen deposition on flower stigmas varied significantly between right target and wrong target visits of a bee species (e.g., *Apis cerana*: Mann-Whitney U = 95, z = − 2.84, *p* < 0.01). More pollen grains were deposited on right target visits than on wrong target visits (Table 4). Stigmatic pollen deposition also differed among the tested bee species (right target visit: Kruskal-Wallis H = 45.54, df = 7, *p* < 0.001; wrong target visit: Kruskal-Wallis H = 15.01, df = 7, *p* < 0.05). *Austronomia ustula*, *Lasioglossum cavernifrons* and *Nomia* (*Hoplonomia*) *elliotii* deposited greater amounts of pollen grains on flower stigmas (Table 4).

**Table 4:** Number of pollen grains deposited on flower stigmas by a single visit of different bee species on *Momordica charantia* flowers.

Bee species	Pollen deposition		Statistical analysis
	Right target visit	Wrong target visit	
*Apis cerana*	268.90^b^ ± 213.43	121.95^ab^ ± 128.25	U = 95, z = -2.84, *p* < 0.01
*Apis dorsata*	276.45^b^ ± 206.82	127.90^ab^ ± 141.56	U = 100, z = -2.71, *p* < 0.01
*Apis florea*	228.05^bc^ ± 199.30	96.30^bc^ ± 111.58	U = 103.50, z = -2.61, *p* < 0.01
*Austronomia ustula*	364.85 ± 240.96	135.35^a^ ± 131.25	U = 76, z = -3.38, *p* < 0.01
*Lasioglossum albescens*	117.17^cd^ ± 82.04	47.10^c^ ± 47.47	U = 85.50, z = -3.10, *p* < 0.01
*Lasioglossum cavernifrons*	366.95^a^ ± 263.0	135.70^a^ ± 134.66	U = 87, z = -3.06, *p* < 0.01
*Lasioglossum funebre*	77.70^d^ ± 54.03	32.60^c^ ± 31.34	U = 85, z = -3.12, *p* < 0.01
*Nomia* (*Hoplonomia*) *elliotii*	382.55^a^ ± 256.02	142.35^a^ ± 146.98	U = 79, z = -3.28, *p* < 0.01
Statistical analysis	Kruskal-Wallis H = 45.54, df = 7, *p* < 0.001	Kruskal-Wallis H = 15.01, df = 7, *p* < 0.001	

Values are given in mean ± standard deviation. Different superscript letters within a column indicate significant differences (Dunn’s post hoc test at 0.05% level).

**Fig. 7:**
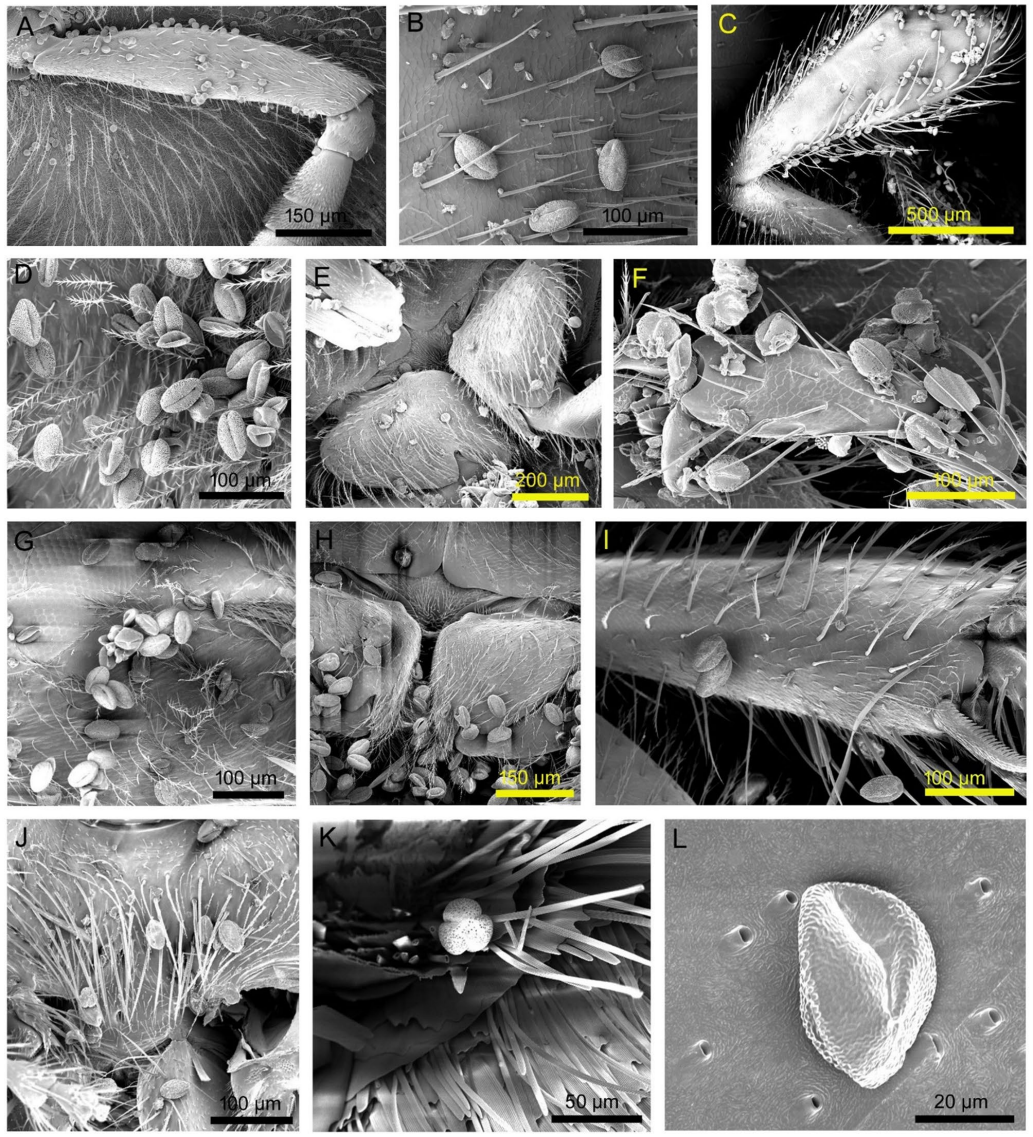
Pollen adhered to the body parts of visitors. (**A**–**C**) *Apis cerana* (head, ventral side of abdomen, and hind leg, respectively), (**D**–**F**) *Lasioglossum cavernifrons* (dorsal side of thorax, ventral side of thorax, and hind leg, respectively), (**G**–**I**) *Lasioglossum funebre* (head, ventral side of thorax, and middle leg, respectively), (**J**) *Tetragonula pagdeni* (ventral side of thorax), (**K**–**L**) *Zizula hylax* (head, and ventral side of abdomen).

### Pollination strategies of flower visitors

Bitter gourd flowers received a greater number of visits from *Apis cerana* (flower visit proportion, FV = 0.131), followed by *Apis florea* (FV = 0.116), *Apis dorsata* (FV = 0.098), *Nomia* (*Hoplonomia*) *elliotii* (FV = 0.056), *Lasioglossum cavernifrons* (FV = 0.052), *Lasioglossum albescens* (FV = 0.050), *Lasioglossum funebre* (FV = 0.049), and *Austronomia ustula* (FV = 0.042) (Table 5).

Coleopteran, dipteran and hemipteran members illegitimately visited the bitter gourd flowers, and hymenopteran and lepidopteran members visited legitimately. During flower visitation by the visitors, pollen adhered to different body parts of the insect (Table 5; Fig. 7). Pollen grains adhered to the head, ventral surface of the thorax and abdomen, and legs of most visitors, including butterflies, honeybees, solitary bees and stingless bees. Pollen grains also adhered to the dorsal surface of bees. The modes of pollination were sternotribic and appendage-mediated for most visitors. However, noto-sternotribic pollination was also noted for honeybees, solitary bees, and stingless bees.

The single-visit pollination efficiency index (PE*i*) was higher in *Apis cerana*, *Austronomia ustula*, *Lasioglossum cavernifrons*, and *Nomia* (*Hoplonomia*) *elliotii*. A comparatively lower single-visit pollination efficiency was recorded for *Apis florea* and *Lasioglossum funebre* (Table 5). The pollination service index (PS*i*) was highest for *Apis cerana* (PS*i* = 0.050), followed by *Apis florea*, *Apis dorsata*, *Lasioglossum cavernifrons*, *Lasioglossum albescens*, *Nomia* (*Hoplonomia*) *elliotii*, *Austronomia ustula*, and *Lasioglossum funebre*.

**Table 5:** Pollination strategies of flower visitors on *Momordica charantia* in West Bengal, India.

Floral visitors	FV	Visitation type	Pollen adhering to body parts	Mode of pollination	AR	SR	PE*i*	PS*i*
• Coleoptera								
*Aulacophora foveicollis*	0.004	IL	-	-	-	**-**	**-**	**-**
*Aulacophora frontalis*	0.005	IL	-	-	-	**-**	**-**	**-**
*Henosepilachna septima*	0.004	IL	-	-	-	**-**	**-**	**-**
*Monolepta signata*	0.003	IL	-	-	-	**-**	**-**	**-**
• Diptera						-		
*Episyrphus balteatus*	0.008	IL	-	-	-	-	-	-
*Helophilus peregrinus*	0.007	IL	-	-	-	-	-	-
*Platycheirus albimanus*	0.005	IL	-	-	-	-	-	-
• Hemiptera								
*Geocoris ochropterus*	0.004	IL	-	-	-	-	-	-
• Hymenoptera								
*Amegilla zonata*	0.048	LV	H, L, DT, VT, VA	S, NS, A	0.95	0.92	-	0.019
*Apis cerana*	0.131	LV	H, L, DT, VT, VA	S, NS, A	0.94	0.90	0.52	0.050
*Apis dorsata*	0.098	LV	H, L, DT, VT, VA	S, NS, A	0.96	0.92	0.45	0.040
*Apis florea*	0.116	LV	H, L, DT, VT, VA	S, NS, A	0.92	0.89	0.40	0.045
*Austronomia ustula*	0.042	LV	H, L, DT, VT, VA	S, NS, A	0.92	0.91	0.55	0.026
*Braunsapis mixta*	0.016	LV	H, L, DT, VT, VA	S, NS, A	0.90	0.89	-	0.007
*Camponotus parius*	0.005	LV	H, L, VT, VA	S, A	-	-	-	-
*Ceratina binghami*	0.020	LV	H, L, DT, VT, VA	S, NS, A	0.92	0.90	-	0.009
*Ceratina hieroglyphica*	0.010	LV	H, L, DT, VT, VA	S, NS, A	0.89	0.88	-	0.004
*Crematogaster laestrygon*	0.006	LV	H, L, VT, VA	S, A	-	-	-	-
*Lasioglossum albescens*	0.050	LV	H, L, DT, VT, VA	S, NS, A	0.90	0.86	0.44	0.029
*Lasioglossum cavernifrons*	0.052	LV	H, L, DT, VT, VA	S, NS, A	0.93	0.90	0.61	0.031
*Lasioglossum funebre*	0.049	LV	H, L, DT, VT, VA	S, NS, A	0.86	0.83	0.43	0.025
*Lasioglossum ovaliceps*	0.007	LV	H, L, DT, VT, VA	S, NS, A	0.85	0.81	-	0.003
*Nomia* (*Hoplonomia*) *elliotii*	0.056	LV	H, L, DT, VT, VA	S, NS, A	0.94	0.93	0.62	0.029
*Nomia* (*Curvinomia*) *strigata*	0.024	LV	H, L, DT, VT, VA	S, NS, A	0.92	0.91	-	0.012
*Scolia soror*	0.007	LV	H, L, VT, VA	S, A	-	-	-	-
*Tetragonula pagdeni*	0.021	LV	H, L, DT, VT, VA	S, NS, A	0.92	0.83	-	0.007
*Thyreus nitidulus*	0.014	LV	H, L, DT, VT, VA	S, NS, A	0.90	0.85	-	0.004
*Trichomyrmex destructor*	0.010	LV	H, L, VT, VA	S, A	-	-	-	-
• Lepidoptera								
*Anthene lycaenina*	0.004	LV	H (p), L, VT, VA	S, A	0.78	0.73	-	0.001
*Appias libythea*	0.007	LV	H (p), L, VT, VA	S, A	0.81	0.74	-	0.002
*Borbo cinnara*	0.009	LV	H (p), L, VT, VA	S, A	-	-	-	-
*Catopsilia pomona*	0.003	LV	H (p), L, VT, VA	S, A	-	-	-	-
*Danaus chrysippus*	0.004	LV	H (p), L, VT, VA	S, A	0.78	0.67	-	0.001
*Euploea core*	0.003	LV	H (p), L, VT, VA	S, A	-	-	-	-
*Eurema blanda*	0.007	LV	H (p), L, VT, VA	S, A	0.85	0.75	-	0.003
*Eurema hecabe*	0.010	LV	H (p), L, VT, VA	S, A	0.86	0.77	-	0.004
*Junonia almana*	0.002	LV	H (p), L, VT, VA	S, A	-	-	-	-
*Junonia atlites*	0.003	LV	H (p), L, VT, VA	S, A	-	-	-	-
*Junonia iphita*	0.002	LV	H (p), L, VT, VA	S, A	-	-	-	-
*Leptosia nina*	0.004	LV	H (p), L, VT, VA	S, A	-	-	-	-
*Papilio polytes*	0.005	LV	H (p), L, VT, VA	S, A	0.72	0.65	-	-
*Pelopidas sinensis*	0.012	LV	H (p), L, VT, VA	S, A	0.91	0.74	-	0.005
*Spoladea recurvalis*	0.004	LV	L, VT, VA	A	-	-	-	-
*Suastus gremius*	0.015	LV	H (p), L, VT, VA	S, A	0.90	0.73	-	0.006
*Zizula hylax*	0.003	LV	H (p), L, VT, VA	S, A	-	-	-	-

Note: FV- flower visit proportion; IV- illegitimate visit, LV- legitimate visit; DA- dorsal side of abdomen, DT- dorsal side of thorax, H- head, H(p)- head (including proboscis), VA- ventral side of abdomen, VT- ventral side of thorax; A- appendage mediated, N- nototribic, NS- noto-sternotribic, S- sternotribic; AR- anther contact rate, SR- stigma contact rate; PE*i*- single-visit pollination efficiency index; PS*i*- pollination service index (derived by multiplication of FV, FS*i*, AR, and SR).

### Fruit and seed sets

No fruits were obtained on the pollinator exclusion treatment (Table 6). In the open pollination system, the fruit set percentage was 81 ± 9.94. After a single visit, the fruit set (%) does not vary significantly among the tested bee species (F_7,72_ = 0.83, *p* = 0.56), ranging from 56.22 ± 9.37 to 64.89 ± 9.75. However, the fruit set differed according to right target and wrong target visits (e.g., *Apis cerana*: t = 3.87, df = 18, *p* < 0.001). The right target visits of pollinators resulted in a greater fruit set than wrong target visits (Table 6). The seed set per flower differed among the eight bee species (Kruskal-Wallis H = 16.52, df = 7, *p* < 0.05). Comparatively higher seed sets were recorded for *Lasioglossum cavernifrons* and *Nomia* (*Hoplonomia*) *elliotii* (Table 6). The seed sets were also varied between right target and wrong target visits (e.g., *Apis cerana*: Mann-Whitney U = 1269.50, z = − 2.06, *p* < 0.05). The right target visits of pollinators resulted in a higher seed set than wrong target visits (Table 6).

**Table 6:** Fruit and seed sets of *Momordica charantia* in different pollination treatments in West Bengal, India.

Pollination treatment	Fruit set (%)	Seed set (seeds/flower)	Statistics
Open pollination	81 ± 9.94	12.33 ± 6.60	
Pollinator exclusion	0	0	
Single-visit on female flowers by pollinators			
*Apis cerana*			
• Right target visit	61.49 ± 10.87	6.35 ± 5.71	Fruit set: t = 3.87, df = 18, ***; Seed set: U = 1269.50, z = − 2.06, *
• Wrong target visit	44 ± 9.27	4.11 ± 4.92	
*Apis dorsata*			
• Right target visit	56.49 ± 13.76	5.49 ± 5.26	Fruit set: t = 2.62, df = 18, *; Seed set: U = 931, z = − 2.01, *
• Wrong target visit	43.67 ± 7.11	3.37 ± 4.14	
*Apis florea*			
• Right target visit	56.22 ± 9.37	4.96 ± 4.84	Fruit set: t = 3.12, df = 18, **; Seed set: U = 1493.50, z = − 2.10, *
• Wrong target visit	42.45 ± 10.34	3.16 ± 3.92	
*Austronomia ustula*			
• Right target visit	61.64 ± 9.20	6.73 ± 5.90	Fruit set: t = 2.64, df = 18, *; Seed set: U = 422.50, z = − 2.03, *
• Wrong target visit	42.50 ± 20.95	3.92 ± 0.88	
*Lasioglossum albescens*			
• Right target visit	60.22 ± 8.60	5.40 ± 5.16	Fruit set: t = 2.60, df = 18, *; Seed set: U = 554, z = − 2.09, *
• Wrong target visit	38.83 ± 24.52	3.00 ± 3.65	
*Lasioglossum cavernifrons*			
• Right target visit	64.89 ± 9.75	7.56 ± 6.14	Fruit set: t = 3.81, df = 18, ***; Seed set: U = 767, z = − 2.18, *
• Wrong target visit	46.67 ± 11.55	4.79 ± 5.72	
*Lasioglossum funebre*			
• Right target visit	60.02 ± 12.95	5.25 ± 4.55	Fruit set: t = 3.07, df = 18, **; Seed set: U = 654, z = − 2.11, *
• Wrong target visit	35.67 ± 21.49	3.16 ± 4.01	
*Nomia* (*Hoplonomia*) *elliotii*			
• Right target visit	63.62 ± 9.54	7.61 ± 6.24	Fruit set: t = 2.95, df = 18, **; Seed set: U = 525.50, z = − 2.23, *
• Wrong target visit	43.33 ± 19.56	4.48 ± 5.55	

Note: *- *p* < 0.05, **-*p* < 0.01, ***-*p* < 0.001.

## Discussion

The male and female flowers of bitter gourd were heteromorphic and reward-biased. The quantities of flower rewards (i.e., nectar and pollen) were higher in male flowers than female flowers (which had only a little amount of nectar). Some researchers (e.g., Oronje et al.^44^) have considered the pistillate flowers of bitter gourd to be rewardless. Considering flower volatile organic compounds (VOCs), both male and female flowers emitted a mixture of many volatile compounds. The composition of VOCs also differed between male and female flowers. These differential floral traits, considering attractants to visitors, between male and female flowers, may lead to a biased preference for flower selection by most visitors.

Numerous insect species from Coleoptera, Diptera, Hemiptera, and Hymenoptera visited bitter gourd flowers. Some researchers (e.g., Bisui et al.^33^, Yogapriya et al.^40^, Balina et al.^45^, Dorjoy et al.^46^) have already documented the floral visitors of the crop species from different regions in India. The visitor composition of a flowering plant changes spatially^47,48^. A few insect species (e.g., *Austronomia ustula*, *Braunsapis mixta*, *Ceratina hieroglyphica*, *Lasioglossum albescens*, *Lasioglossum ovaliceps*, and *Scolia soror*) were newly recognised as floral visitors of bitter gourd. Abundant visitors were honeybees (*Apis* spp.) and solitary bees (e.g., *Lasioglossum cavernifrons*, *Lasioglossum funebre*, and *Nomia* (*Hoplonomia*) *elliotii*). The abundance of honeybees on bitter gourd was also reported from other regions^39,45,46^. However, some researchers have reported other abundant bee species, such as *Megachile lanata*^45^ and *Tetragonula iridipennis*^40^. Landscape composition largely influences flower visitors’ abundance, richness, and diversity^49–51^, as it determines the availability of nesting sites, food resources, and interactions. Visitors’ abundance, richness, and diversity in bitter gourd changed according to daytime, with higher values during 6:00–10:00 h. Temporal variations of these parameters are well-recognized in many plant species^51,52^. Visitors’ activity and the availability of floral rewards change with daytime and may remain optimum at mid-morning.

Cucurbitaceae crop plants are staminate flower-biased. The flower sex ratio of cucurbits also influences the pollination efficiency of the visitors^53^. The flower sex selection index (FS*i*) was less than 1 for most visitors. That means most insect visitors preferred male flowers over female flowers. The male-biased flower selections of visitors were governed by multiple floral traits, including flower morphology (e.g., flower size and colour), floral resources (e.g., nectar and pollen availability), and flower volatile organic compounds (male and female flowers had a mixture of VOCs of dissimilar composition). Beetles fed on floral tissues; some bees collected nectar and pollens, and others collected only nectar. Beetles as florivores on Cucubitaceae are well documented by several researchers (e.g., Fronk and Slater^54^, Gardner et al.^55^, Layek et al.^28^). Bees having corbiculae or scopae are mostly mixed foragers (collect both nectar and pollen grains); bugs, butterflies, moths, and wasps commonly collect only nectar. The type of floral resources a visitor can collect depends on the available floral rewards, the visitor’s morphometry, foraging behaviour, and the visitor’s demands.

Flower visitors conducted the right target visits (i.e., collected floral resources during the visit) and sometimes wrong target visits without collecting floral resources. The phenomenon of wrong target visits is reported for the first time by us. The conduction of wrong target visits is due to the visitors’ inability to differentiate between resource-filled and resource-less flowers from a reasonable distance. Female flowers got more wrong target visits than male flowers. This is due to a lower amount of floral resources within female flowers compared to male flowers.

The flower visitation rate remained higher for honeybees and solitary bees and lower for lepidopteran members and stingless bees. Saeed et al.^39^, from Multan, Pakistan, reported a slightly lower visitation rate for bees on bitter gourd. The visitation rate of visitors depends on floral arrangement, density, and the availability and accessibility of floral resources. In the early morning, the availability of floral resources was high, and the flower handling time was comparatively higher. So, most insect visitors’ visitation rate was lower in the early morning. In the late afternoon, the reduced accessibility of resources leads to increased flower handling time and a decreased visitation rate.

Pollen deposition efficiencies varied among the bee species, with higher rates for *Austronomia ustula*, *Lasioglossum cavernifrons* and *Nomia* (*Hoplonomia*) *elliotii*. Pollen transfer on flower stigma depends on the morphometry of the bee species, resource collection patterns and foraging behaviour. Pollinators deposited more pollen grains on the right target visits than on the wrong target visits. This could be due to two reasons. First, they touched stigma more frequently on right target visits than on wrong target visits (about 49.13 ± 9.86% of legitimate visits). Second, pollinators spent less time on a wrong target visit than on a right target visit. Many researchers (e.g., Ne’eman et al.^56^, Willmer et al.^57^) have used pollen deposition on the stigma after single visits to estimate pollinator effectiveness. However, Wang et al.^58^ observed weak correlations between pollen deposition on stigma and seed production.

The flower visitor’s body parts, which were covered with pollens, were the head parts, the dorsal and ventral sides of the thorax, the ventral side of the abdomen, and the legs. The pollen attachment sites indicate the pollen transferring (i.e., pollinating) body parts of the visitors. Most visitors pollinated bitter gourd flowers sternotribically, using the ventral surface of their thorax and abdomen. The mode of pollination depends on flower morphology and pollen deposition patterns on the visitor’s body^59,60^. Excluding labiate flowers, other floral morphs (e.g. funnel-shaped, dish-shaped, or saucer-shaped) facilitate sternotribic pollination. However, most bees (e.g., honeybees, solitary bees, and stingless bees) exhibited a noto-sternotribic mode of pollination. Appendage-mediated pollination was also prevalent among most pollinator species visiting bitter gourd flowers with exposed stigmatic surfaces. The single-visit pollination efficiency index (PE*i*) remained higher for *Apis cerana*, *Austronomia ustula*, *Lasioglossum cavernifrons* and *Nomia* (*Hoplonomia*) *elliotii*. However, the effectiveness of a pollinator species depends not only on single-visit pollination efficiencies but also on multiple parameters, including its abundance on the plant species, its flower visitation rate, and its foraging strategies. In this regard, we estimated a combined index, the pollination service index (PS*i*), by considering multiple parameters, including the proportion of flower visits, the flower sex selection index, the anther contact rate, and the stigma contact rate. Based on PS*i* values, effective pollinators were honeybees (*Apis* spp.) and solitary bees (*Austronomia ustula*, *Lasioglossum* spp. and *Nomia* (*Hoplonomia*) *elliotii*). Some researchers (e.g., Balina et al.^45^, Subhankar et al.^38^, Yogapriya et al.^40^) have previously established the vital role of honeybees and solitary bees in bitter gourd pollination. The morphometry of bees, their foraging behaviour (making more bodily contact with sex organs and high visitation rate) and their abundance determine their importance as pollinators^21,58,61^.

The fruit and seed sets of bitter gourd obligatorily depended on pollinators’ visits. The results of pollinator exclusion treatments highlight the dependency of the crop species on pollinators for reproduction. Cucurbits commonly experience complete fruit loss in the absence of pollinators^9,30^. The right target and wrong target visits of pollinators resulted in fruit and seed sets. The production of fruit and seeds remained lower for wrong target single-visit treatments than for right target single-visit treatments. Female flowers received many wrong target visits (about 43.79 ± 12.38%) by pollinators; this type of visit has provided an additional pollination service to bitter gourd and enhanced the reproductive success of the plant species.

## Conclusions

The differential flower traits (including attractants such as size, colour, and flower rewards, as well as flower volatile organic compounds) of bitter gourd raised biases in male and female flower selection by visitors. Diverse insects (e.g., beetles, butterflies, flies, honeybees, moths, solitary bees, stingless bees, and wasps) visited bitter gourd flowers. Abundant floral visitors were *Apis cerana*, *Apis dorsata*, *Apis florea*, *Austronomia ustula*, *Lasioglossum albescens*, *Lasioglossum cavernifrons*, *Lasioglossum funebre*, and *Nomia* (*Hoplonomia*) *elliotii*. All of these were effective pollinators of bitter gourd, and the pollination service index (PS*i*) value was highest for *Apis cerana*, followed by *Apis florea*, *Apis dorsata*, *Lasioglossum cavernifrons*, *Lasioglossum albescens*, *Nomia* (*Hoplonomia*) *elliotii*,* Austronomia ustula*, and *Lasioglossum funebre*. The pollinators carried out the right target and the wrong target visits on female flowers. In addition to the right target visits, wrong target visits delivered substantial pollen grains on flower stigmas, resulting in fruit and seed sets. Therefore, it can be concluded that wrong target flower visits on bitter gourd have a vital role in plant reproduction.

## Supplementary Information

Below is the link to the electronic supplementary material.

Supplementary Material 1

Supplementary Material 2

Supplementary Material 3

Supplementary Material 4

Supplementary Material 5

Supplementary Material 6

Supplementary Material 7

Supplementary Material 8

## Data Availability

Data is provided within the manuscript or supplementary information files.
