# Supplementary material for: Interactions of flower visitors with bitter gourd (Momordica charantia L.) and effects of right target and wrong target flower visits on plant reproduction
Source: Sci Rep. 2025 Oct 22;15:36974. doi: 10.1038/s41598-025-20968-w (PMC12546850; doi:10.1038/s41598-025-20968-w)
Supplement: Supplementary file 1 — Supplementary Material 1 [file 41598_2025_20968_MOESM1_ESM.docx]

**Table S1.** The methodologies for estimating some flower traits (flower shape, size, colour, pollen content, nectar content, and volatile organic compounds) of *Momordica charantia* in West Bengal, India.

| Traits | Different steps |
| --- | --- |
| Flower shape | 1. We observed a few male and female flowers (n = 10 for each category) and recorded the shape of the flowers. |
| Flower size | 1. We observed male and female flowers (n = 10 for each category) and recorded the diameter of flowers using a millimetre scale. |
| Flower colour | 1. We observed male and female flowers (n = 10 for each category) and recorded flower colour with naked human eyes. |
| Pollen content/male flower | 1. We selected mature flower buds (n = 10) before the flowers opened, between 2:00 and 3:00 a.m., and collected the anthers separately into 10 vials. 2. Then, we added 70% ethanol (< 1 mL). 3. Then, we crushed the anthers and added ethanol to adjust the volume to 1 mL. 4. We took 10 µL of pollen solution on a clean slide and counted the number of pollen grains. We repeated this process three times for a sample (derived from a male flower anther), and an average value was taken. 5. Then, multiply the average value (i.e., number of pollen grains) by 100 to transform the value for 1 mL (= one male flower) |
| Nectar content/ flower | 1. Mature flower buds were selected (n = 10 male flower buds, n = 10 female flower buds) and bagged with nylon net from 2:00 to 3:00 h to restrict the visitation of insects. 2. Nectar collected with a microcapillary tube at two-hour intervals. During collection, flowers opened, slightly ruptured calyx to open nectarines. Then, touched the stored nectar with the tip of the capillary tube. After collection, flowers were re-bagged. 3. Nectar collection continued for the entire longevity period of the flowers. The total amount of nectar collected by an individual flower was recorded. |
| Flower volatile organic compounds (VOCs) | 1. Flowers (4–5 male flowers; 4–5 female flowers) were taken into a 20 mL glass vial (Thermo Fisher Scientific), capped and incubated for 3–4 h at room temperature. 2. The vials were loaded into the TriPlus RSH autosampler, which used a splitless injector to inject the volatile sample into the Trace 1300 gas chromatography (Thermo Fisher Scientific). 3. A TG-WAX MS column (Thermo Fisher Scientific) with helium as carrier gas (flow rate: 1.20 mL/min) was used to separate volatile compounds in the GC system. 4. The temperature was set to 60°C for 2 min and then increased to 250°C (10°C/min), keeping the transfer line temperature at 250°C. 5. The GC was linked to an ISQ QD single Quadrupole Mass Spectrometer (Thermo Fisher Scientific). The mass was scanned with a range of 40-400 a.m.u. and at 70 eV. 6. We tentatively identified the VOCs by comparing their mass and retention indices (RIs) to those in the NIST 2017 library. |
