# Supplementary material for: Interactions of flower visitors with bitter gourd (Momordica charantia L.) and effects of right target and wrong target flower visits on plant reproduction
Source: Sci Rep. 2025 Oct 22;15:36974. doi: 10.1038/s41598-025-20968-w (PMC12546850; doi:10.1038/s41598-025-20968-w)
Supplement: Supplementary file 2 — Supplementary Material 2 [file 41598_2025_20968_MOESM2_ESM.docx]

**Table S2.** Sample sizes (number of flowers) to determine stigmatic pollen deposition, fruit, and seed sets for different treatments and bee species on *Momordica charantia* in West Bengal, India.

| Treatment | Total number of encountered flowers | No. of flowers utilised in determining pollen deposition | No. of flowers utilised in determining fruit and seed sets |
| --- | --- | --- | --- |
| Open pollination | 100 | - | 100 |
| Pollinator exclusion | 50 | - | 50 |
| Single-visit experiment |  |  |  |
| *Apis cerana* |  |  |  |
| - Right targeted visit | 92 | 20 | 72 |
| - Wrong targeted visit | 65 | 20 | 45 |
| *Apis dorsata* |  |  |  |
| - Right targeted visit | 85 | 20 | 65 |
| - Wrong targeted visit | 57 | 20 | 37 |
| *Apis florea* |  |  |  |
| - Right targeted visit | 97 | 20 | 77 |
| - Wrong targeted visit | 69 | 20 | 49 |
| *Austronomia ustula* |  |  |  |
| - Right targeted visit | 65 | 20 | 45 |
| - Wrong targeted visit | 46 | 20 | 26 |
| *Lasioglossum albescens* |  |  |  |
| - Right targeted visit | 67 | 20 | 47 |
| - Wrong targeted visit | 52 | 20 | 32 |
| *Lasioglossum cavernifrons* |  |  |  |
| - Right targeted visit | 81 | 20 | 61 |
| - Wrong targeted visit | 54 | 20 | 34 |
| *Lasioglossum funebre* |  |  |  |
| - Right targeted visit | 77 | 20 | 57 |
| - Wrong targeted visit | 51 | 20 | 31 |
| *Nomia* (*Hoplonomia*) *elliotii* |  |  |  |
| - Right targeted visit | 71 | 20 | 51 |
| - Wrong targeted visit | 49 | 20 | 29 |
