# Supplementary material for: Interactions of flower visitors with bitter gourd (Momordica charantia L.) and effects of right target and wrong target flower visits on plant reproduction
Source: Sci Rep. 2025 Oct 22;15:36974. doi: 10.1038/s41598-025-20968-w (PMC12546850; doi:10.1038/s41598-025-20968-w)
Supplement: Supplementary file 3 — Supplementary Material 3 [file 41598_2025_20968_MOESM3_ESM.docx]

**Table S3.** Volatile organic compounds (VOCs) in male and female flowers of *Momordica charantia*.

| VOCs | Male flowers | Female flowers |
| --- | --- | --- |
| 1-(2-Acetoxyethyl)-3,6-diazahomoadamantan-9-one oxime | + | + |
| 2-(16-Acetoxy-11-hydroxy-4, 8,10,14-tetramethyl-3-oxohexadecahydrocyclopenta [a] phenanthren-17-ylidene)-6-methyl hept-5-enoic acid, methyl ester | ++ |  |
| Aconitane-1,7,8,14-tetrol, 20-ethyl-6,16-dimethoxy-4-(methoxymethyl)-, 14-acetate, (1à,6á,14à,16á) | + |  |
| Androstane-11,17-dione, 3-[(trimethylsilyl)oxy]-, 17-[O-(phenylmethyl)oxime], (3à,5à) | + |  |
| 6-Azacholest-4-en-7-one, 6-benzyl-3à-hydroxy | + | + |
| Benzyl Benzoate |  | + |
| 1,1'-Biphenyl, 3,4-diethyl- |  | ++ |
| 3-[3-Bromophenyl]-7-chloro-3,4-dihydro-10-hydroxy-1,9(2H,10H)-acridinedione |  | ++ |
| 2-Bromotetradecanoic acid |  | + |
| 3-Butoxy-1,1,1,7,7,7-hexamethyl-3,5,5 tris(trimethylsiloxy) tetrasiloxane |  | + |
| Cetene | + | + |
| 24-Chloro-25-methoxylanost-8-en-3-yl acetate | ++ |  |
| β-Copaene | +++ |  |
| Cyclohexasiloxane, dodecamethyl- |  | +++ |
| Cyclotrisiloxane, hexamethyl- | ++ |  |
| 9-Desoxo-9-x-acetoxy-3,8,12-tri-O-acetylingol | + | ++ |
| Diethyl Phthalate |  | + |
| 17-(1,5-Dimethylhexyl)-10,13-dimethyl-3-styryl hexadecahydrocyclopenta [a] phenanthren-2-one | +++ | ++ |
| Distearin |  | + |
| 7,8-Epoxylanostan-11-ol, 3-acetoxy- | ++ | + |
| Ethyl iso-allocholate | + | +++ |
| Hematoporphyrin | +++ |  |
| Hexasiloxane, 1,1,3,3,5,5,7,7,9,9,11,11-dodecamethyl- | +++ | +++ |
| 5H-Cyclopropa[3,4]benz[1,2 e]azulen-5-one, 3,9,9a-tris(acetyloxy)-3-[(acet yloxy)methyl]-2-chloro-1,1a,1 b,2,3,4,4a,7a,7b,8,9,9a-dodec ahydro-4a,7b-dihydroxy-1,1,6,8-tetramethyl-,[1aR (1aà,1bá,2à,3á,4aá,7aà,7 bà,8à,9á,9aà)] | + |  |
| 3'H-Cycloprop (1,2) cholesta-1,4,6-trien-3-one, 1'-carboethoxy-1'-cyano-1á,2á-dihydro | +++ | + |
| Hexasiloxane, 1,1,3,3,5,5,7,7,9,9,11,11-dodecamethyl | + | + |
| 1H-Indene, 1-methylene- |  | + |
| 3-[18-(3-Hydroxy-propyl)-3,3,7,12,17-pentamethyl-2,3,22,24-tetrahydro-porphin-2-yl]propan-1-ol | + | + |
| Lycopene, 1,1',2,2'-tetrahydro-1,1'-dimethoxy-, all-trans- | +++ | + |
| Milbemycin B,6,28-anhydro-15-chloro-25-isopropyl-13-dehydro-5-O-demethyl-4-methyl- | ++ | + |
| Morphinan-4,5-epoxy-3,6-di-ol, 6-[7-nitrobenzofurazan-4-yl]amino- | + | +++ |
| Octadecane, 3-ethyl-5-(2-ethylbutyl)- |  | + |
| 9-Octadecenoic acid, (2-phenyl-1,3-dioxolan-4-yl)methyl ester, cis- |  | + |
| Octasiloxane, 1,1,3,3,5,5,7,7,9,9,11,11,13,13,15,15-hexadecamethyl- |  | + |
| Olean-12-ene-3,15,16,21,22,28-hexol, (3á,15à,16à,21á,22à) | + |  |
| (5á)Pregnane-3,20á-diol, 14à,18à-[4-methyl-3-oxo-(1-oxa-4-azabutane-1,4-diyl)]-, diacetate | + | + |
| Pseudosolasodine diacetate |  | ++ |
| Rhodopin | + | ++ |
| L-Valine, N-[N,O-bis(2,4-dinitrophenyl)-L-tyrosyl]-, methyl ester | ++ | ++ |

Note: +++ highly abundant, ++ moderate abundance, + less abundant.
