# Supplementary material for: Interactions of flower visitors with bitter gourd (Momordica charantia L.) and effects of right target and wrong target flower visits on plant reproduction
Source: Sci Rep. 2025 Oct 22;15:36974. doi: 10.1038/s41598-025-20968-w (PMC12546850; doi:10.1038/s41598-025-20968-w)
Supplement: Supplementary file 4 — Supplementary Material 4 [file 41598_2025_20968_MOESM4_ESM.docx]

**Table S4.** Floral visitors of *Momordica charantia* in West Bengal, India.

| Insect order | Family | Group | Insect species |
| --- | --- | --- | --- |
| Coleoptera | Chrysomelidae | Beetle | *Aulacophora foveicollis*, *Aulacophora frontalis*, *Monolepta signata* |
|  | Coccinellidae | Beetle | *Henosepilachna septima* |
| Diptera | Syrphidae | Fly | *Episyrphus balteatus*, *Helophilus peregrinus*, *Platycheirus albimanus* |
| Hemiptera | Geocorinae | Bug | *Geocoris ochropterus* |
| Hymenoptera | Apidae | Bee | *Amegilla zonata*, *Apis cerana*, *Apis dorsata*, *Apis florea*, *Braunsapis mixta*, *Tetragonula pagdeni*, *Thyreus nitidulus* |
|  | Formicidae | Ant | *Camponotus parius, Crematogaster laestrygon, Trichomyrmex destructor* |
|  | Halictidae | Bee | *Austronomia ustula*, *Ceratina binghami*, *Ceratina hieroglyphica*, *Lasioglossum albescens*, *Lasioglossum cavernifrons*, *Lasioglossum funebre*, *Lasioglossum ovaliceps, Nomia* (*Curvinomia*) *strigata*, *Nomia* (*Hoplonomia*) *elliotii* |
|  | Scoliidae | Wasp | *Scolia soror* |
| Lepidoptera | Crambidea | Moth | *Spoladea recurvalis* |
|  | Hesperiidae | Butterfly | *Borbo cinnara,* *Pelopidas sinensis*, *Suastus gremius* |
|  | Lycaenidae | Butterfly | *Anthene lycaenina*, *Zizula hylax* |
|  | Nymphalidae | Butterfly | *Danaus chrysippus*, *Euploea core*, *Junonia almana*, *Junonia atlites*, *Junonia iphita* |
|  | Papilionidae | Butterfly | *Papilio polytes* |
|  | Pieridae | Butterfly | *Appias libythea*, *Catopsilia pomona*, *Eurema blanda*, *Eurema hecabe*, *Leptosia nina* |
