# Supplementary material for: Interactions of flower visitors with bitter gourd (Momordica charantia L.) and effects of right target and wrong target flower visits on plant reproduction
Source: Sci Rep. 2025 Oct 22;15:36974. doi: 10.1038/s41598-025-20968-w (PMC12546850; doi:10.1038/s41598-025-20968-w)
Supplement: Supplementary file 5 — Supplementary Material 5 [file 41598_2025_20968_MOESM5_ESM.docx]

**Table S5.** Daytime-wise abundance (number of visitors/m^2^ area/5 min) of floral visitors of *Momordica charantia* in West Bengal, India.

| Visitor | Daytime-wise abundance | | | | | | | Statistical analysis |
| --- | --- | --- | --- | --- | --- | --- | --- | --- |
|  | 4.00–6.00 h | 6.00–8.00 h | 8.00–10.00 h | 10.00–12.00 h | 12.00–14.00 h | 14.00–16.00 h | 16.00–18.00 h |  |
| *Apis cerana* | 0.25^c^ ± 0.67 | 1.30^a^ ± 1.40 | 1.28^a^ ± 1.57 | 1.08^ab^ ± 1.51 | 0.72^b^± 1.09 | 0.20^c^ ± 0.46 | 0.08^d^ ± 0.27 | χ^2^ = 39.95, df = 6, p<0.001 |
| *Apis dorsata* | 0.18^c^ ± 0.50 | 0.90^a^ ± 1.15 | 0.85^a^ ± 1.27 | 0.70^ab^ ± 1.04 | 0.48^b^ ± 0.88 | 0.15^c^ ± 0.43 | 0.10^c^ ± 0.30 | χ^2^ = 32.19, df = 6, p<0.001 |
| *Apis florea* | 0.12^d^ ± 0.46 | 1.65^a^ ± 1.58 | 1.42^ab^ ± 1.60 | 1.25^b^ ± 1.71 | 0.58^c^ ± 0.98 | 0.18^d^ ± 0.38 | 0.08^e^ ± 0.27 | χ^2^ = 54.32, df = 6, p<0.001 |
| *Austronomia ustula* | 0.38^ab^ ± 0.81 | 0.50^a^ ± 0.88 | 0.40^ab^ ± 0.90 | 0.35^ab^ ± 0.70 | 0.22^b^ ± 0.58 | 0.12^c^ ± 0.33 | 0.05^c^ ± 0.22 | χ^2^ = 11.07, df = 6, p<0.05 |
| *Lasioglossum albescens* | 0.50^ab^ ± 0.82 | 0.65^a^ ± 1.08 | 0.55^ab^ ± 0.99 | 0.42^b^ ± 0.84 | 0.30^bc^ ± 0.61 | 0.15^c^ ± 0.36 | 0.08^c^ ± 0.27 | χ^2^ = 12.89, df = 6, p<0.05 |
| *Lasioglossum cavernifrons* | 0.78^ab^ ± 1.17 | 0.95^a^ ± 1.20 | 0.80^ab^ ± 1.11 | 0.50^b^ ± 1.04 | 0.30^bc^ ± 0.69 | 0.10^c^ ± 0.38 | 0.05^c^ ± 0.22 | χ^2^ = 34.55, df = 6, p<0.001 |
| *Lasioglossum funebre* | 0.12^bc^ ± 0.46 | 0.68^a^ ± 0.92 | 0.72^a^ ± 0.93 | 0.65^a^ ± 0.92 | 0.30^b^ ± 0.61 | 0.18^bc^ ± 0.45 | 0.08^c^ ± 0.27 | χ^2^ = 35.29, df = 6, p<0.001 |
| *Nomia* (*Hoplonomia*) *elliotii* | 0.10^c^ ± 0.30 | 0.68^a^ ± 1.05 | 0.62^a^ ± 0.95 | 0.40^b^ ± 0.67 | 0.18^c^ ± 0.45 | 0.08^cd^ ± 0.27 | 0.02^cd^ ± 0.16 | χ^2^ = 30.83, df = 6, p<0.001 |

Values are given in mean ± standard deviation. Different superscript letters within a row (followed by mean values) indicate significant differences (Kruskal-Wallis test followed by Dunn’s post hoc test, 0.05%).
