# Supplementary material for: Interactions of flower visitors with bitter gourd (Momordica charantia L.) and effects of right target and wrong target flower visits on plant reproduction
Source: Sci Rep. 2025 Oct 22;15:36974. doi: 10.1038/s41598-025-20968-w (PMC12546850; doi:10.1038/s41598-025-20968-w)
Supplement: Supplementary file 6 — Supplementary Material 6 [file 41598_2025_20968_MOESM6_ESM.docx]

**Table S6.** Daytime-wise, insect visits received a flower (number of visits/flower/5 min) of *Momordica charantia* in West Bengal, India.

| Daytime | Insect visits/flower/5 min | |
| --- | --- | --- |
|  | Male flower | Female flower |
| 4.00–6.00 h | 1.05^d^ ± 1.15 | 0.75^bcd^ ± 0.85 |
| 6.00–8.00 h | 2.80^a^ ± 1.58 | 1.85^a^ ± 1.31 |
| 8.00–10.00 h | 2.30^ab^ ± 1.03 | 1.60^a^ ± 1.10 |
| 10.00–12.00 h | 1.75^bc^ ± 1.12 | 1.35^ab^ ± 1.04 |
| 12.00–14.00 h | 1.20^cd^ ± 1.01 | 0.95^bc^ ± 1.00 |
| 14.00–16.00 h | 0.55^de^ ± 0.69 | 0.40^cd^ ± 0.60 |
| 16.00–18.00 h | 0.20^e^ ± 0.41 | 0.15^d^ ± 0.37 |
| Statistical analysis | H = 59.03, df = 6, p<0.001 | H = 38.97, df = 6, p<0.001 |

Values are given in mean ± standard deviation. Different superscript letters within a column (followed by mean values) indicate significant differences (Kruskal-Wallis test followed by Dunn’s post hoc test, 0.05%).
