# Supplementary material for: Interactions of flower visitors with bitter gourd (Momordica charantia L.) and effects of right target and wrong target flower visits on plant reproduction
Source: Sci Rep. 2025 Oct 22;15:36974. doi: 10.1038/s41598-025-20968-w (PMC12546850; doi:10.1038/s41598-025-20968-w)
Supplement: Supplementary file 7 — Supplementary Material 7 [file 41598_2025_20968_MOESM7_ESM.docx]

**Table S7.** Daytime-wise flower visitation rate (number of flowers visited/ min) of floral visitors of *Momordica charantia* in West Bengal, India.

| Visitor | Daytime-wise visitation rate | | | | | | | Statistical analysis |
| --- | --- | --- | --- | --- | --- | --- | --- | --- |
|  | 4.00–6.00 h | 6.00–8.00 h | 8.00–10.00 h | 10.00–12.00 h | 12.00–14.00 h | 14.00–16.00 h | 16.00–18.00 h |  |
| *Apis cerana* | 13.70^ab^ ± 3.18 | 15.25^a^ ± 2.83 | 16.35^a^ ± 3.15 | 17.05^a^ ± 3.19 | 15.95^a^± 2.89 | 14.10^ab^ ± 2.99 | 13.45^b^ ± 3.24 | χ^2^ = 18.54, df = 6, p<0.01 |
| *Apis dorsata* | 12.95^ab^ ± 3.69 | 14.30^a^ ± 3.45 | 15.30^a^ ± 3.73 | 15.85^a^ ± 3.38 | 14.40^a^ ± 3.10 | 13.05^ab^ ± 3.03 | 12.30^b^ ± 3.31 | χ^2^ = 14.33, df = 6, p<0.05 |
| *Apis florea* | 6.40^b^ ± 1.88 | 6.75^ab^ ± 1.45 | 8.10^a^ ± 2.38 | 8.80^a^ ± 2.28 | 7.15^ab^ ± 1.81 | 6.00^bc^ ± 1.89 | 5.65^bc^ ± 1.84 | χ^2^ = 27.73, df = 6, p<0.001 |
| *Austronomia ustula* | 5.40^b^ ± 1.60 | 5.70^ab^ ± 1.72 | 6.45^a^ ± 1.61 | 6.75^a^ ± 1.86 | 5.80^ab^ ± 1.58 | 5.35^b^ ± 1.35 | 5.00^b^ ± 1.62 | χ^2^ = 14.02, df = 6, p<0.05 |
| *Lasioglossum albescens* | 5.65^b^ ± 1.42 | 6.10^ab^ ± 1.68 | 6.80^a^ ± 1.91 | 7.10^a^ ± 2.97 | 6.10^ab^ ± 1.45 | 5.50^b^ ± 1.28 | 5.15^b^ ± 1.63 | χ^2^ = 17.10, df = 6, p<0.01 |
| *Lasioglossum cavernifrons* | 4.20^b^ ± 1.70 | 4.60^a^ ± 1.90 | 5.70^a^ ± 1.98 | 6.15^a^ ± 1.90 | 5.05^ab^ ± 1.70 | 4.55^ab^ ± 1.73 | 3.70^bc^ ± 1.38 | χ^2^ = 22.14, df = 6, p<0.01 |
| *Lasioglossum funebre* | 5.45^ab^ ± 1.64 | 5.70^ab^ ± 1.66 | 6.50^a^ ± 2.06 | 7.00^a^ ± 2.32 | 6.05^ab^ ± 2.24 | 5.25^b^ ± 1.68 | 5.10^b^ ± 1.65 | χ^2^ = 12.91, df = 6, p<0.05 |
| *Nomia* (*Hoplonomia*) *elliotii* | 6.65^ab^ ± 2.01 | 7.15^ab^ ± 2.06 | 8.10^a^ ± 2.20 | 8.45^a^ ± 2.14 | 7.55^a^ ± 2.06 | 6.90^ab^ ± 2.07 | 6.75^ab^ ± 2.24 | χ^2^ = 12.98, df = 6, p<0.05 |

Values are given in mean ± standard deviation. Different superscript letters within a row (followed by mean values) indicate significant differences (Kruskal-Wallis test followed by Dunn’s post hoc test, 0.05%).
