# Supplementary material for: Interactions of flower visitors with bitter gourd (Momordica charantia L.) and effects of right target and wrong target flower visits on plant reproduction
Source: Sci Rep. 2025 Oct 22;15:36974. doi: 10.1038/s41598-025-20968-w (PMC12546850; doi:10.1038/s41598-025-20968-w)
Supplement: Supplementary file 8 — Supplementary Material 8 [file 41598_2025_20968_MOESM8_ESM.docx]

**Table S8.** Daytime-wise flower handling time (time spent per visit on a flower) of floral visitors of *Momordica charantia* in West Bengal, India.

| Visitor | Flower handling time (sec) | | | | | | | Statistical analysis |
| --- | --- | --- | --- | --- | --- | --- | --- | --- |
|  | 4.00–6.00 h | 6.00–8.00 h | 8.00–10.00 h | 10.00–12.00 h | 12.00–14.00 h | 14.00–16.00 h | 16.00–18.00 h |  |
| *Apis cerana* | 9.30^a^ ± 4.99 | 8.21^a^ ± 4.29 | 6.09^b^ ± 3.52 | 5.36^bc^ ± 2.43 | 4.97^c^ ± 2.21 | 5.75^b^ ± 3.29 | 6.49^b^ ± 3.68 | χ^2^ = 19.54, df = 6, p<0.01 |
| *Apis dorsata* | 13.38^a^ ± 8.98 | 10.62^ab^ ± 7.24 | 8.18^b^ ± 4.39 | 7.07^b^ ± 3.95 | 6.37^b^ ± 3.15 | 7.37^b^ ± 4.39 | 7.75^b^ ± 4.66 | χ^2^ = 15.33, df = 6, p<0.05 |
| *Apis florea* | 12.81^a^ ± 10.24 | 10.09^ab^ ± 9.28 | 7.99^b^ ± 7.10 | 6.97^b^ ± 5.76 | 6.34^bc^ ± 4.57 | 7.82^b^ ± 5.43 | 8.44^ab^ ± 5.90 | χ^2^ = 15.01, df = 6, p<0.05 |
| *Austronomia ustula* | 10.40^a^ ± 5.47 | 8.14^ab^ ± 3.75 | 6.72^b^ ± 2.88 | 6.05^b^ ± 2.60 | 5.96^b^ ± 2.49 | 6.89^b^ ± 2.52 | 7.26^ab^ ± 2.95 | χ^2^ = 16.33, df = 6, p<0.05 |
| *Lasioglossum albescens* | 9.91^a^ ± 5.69 | 7.23^a^ ± 3.65 | 5.98^a^ ± 2.94 | 5.57^ab^ ± 2.18 | 5.40^ab^ ± 2.47 | 6.16^ab^ ± 2.81 | 6.45^a^ ± 3.03 | χ^2^ = 14.67, df = 6, p<0.05 |
| *Lasioglossum cavernifrons* | 18.58^a^ ± 18.86 | 16.15^a^ ± 15.31 | 12.58^ab^ ± 10.58 | 10.66^b^ ± 9.73 | 10.14^b^ ± 11.43 | 10.52^b^ ± 8.32 | 10.80^b^ ± 7.32 | χ^2^ = 15.60, df = 6, p<0.05 |
| *Lasioglossum funebre* | 11.52^a^ ± 7.74 | 9.19^ab^ ± 5.04 | 7.05^b^ ± 3.50 | 6.29^b^ ± 2.79 | 6.02^b^ ± 3.05 | 6.41^b^ ± 2.71 | 6.86^b^ ± 3.16 | χ^2^ = 14.91, df = 6, p<0.05 |
| *Nomia* (*Hoplonomia*) *elliotii* | 10.71^a^ ± 5.58 | 8.91^ab^ ± 4.62 | 7.08^ab^ ± 2.76 | 6.44^b^ ± 2.97 | 6.17^b^ ± 2.61 | 6.85^b^ ± 2.98 | 7.34^ab^ ± 3.09 | χ^2^ = 14.11, df = 6, p<0.05 |

Values are given in mean ± standard deviation. Different superscript letters within a row (followed by mean values) indicate significant differences (Kruskal-Wallis test followed by Dunn’s post hoc test, 0.05%).
